# Expression of RUNX1-JAK2 in Human Induced Pluripotent Stem Cell-Derived Hematopoietic Cells Activates the JAK-STAT and MYC Pathways

**DOI:** 10.3390/ijms22147576

**Published:** 2021-07-15

**Authors:** Klaus Fortschegger, Anna-Maria Husa, Dagmar Schinnerl, Karin Nebral, Sabine Strehl

**Affiliations:** St. Anna Children’s Cancer Research Institute (CCRI), 1090 Vienna, Austria; anna-maria.husa@ccri.at (A.-M.H.); dagmar.schinnerl@ccri.at (D.S.); karin.nebral@labdia.at (K.N.); sabine.strehl@ccri.at (S.S.)

**Keywords:** leukemia, oncogenic fusion, CRISPR/Cas9, hematopoiesis, hiPSC, JAK-STAT signaling, MYC pathway

## Abstract

A heterogeneous genetic subtype of B-cell precursor acute lymphoblastic leukemia is driven by constitutive kinase-activation, including patients with JAK2 fusions. In our study, we model the impact of a novel JAK2 fusion protein on hematopoietic development in human induced pluripotent stem cells (hiPSCs). We insert the *RUNX1-JAK2* fusion into one endogenous *RUNX1* allele through employing in trans paired nicking genome editing. Tagging of the fusion with a degron facilitates protein depletion using the heterobifunctional compound dTAG-13. Throughout in vitro hematopoietic differentiation, the expression of RUNX1-JAK2 is driven by endogenous *RUNX1* regulatory elements at physiological levels. Functional analysis reveals that *RUNX1-JAK2* knock-in cell lines yield fewer hematopoietic progenitors, due to *RUNX1* haploinsufficiency. Nevertheless, these progenitors further differentiate toward myeloid lineages to a similar extent as wild-type cells. The expression of the RUNX1-JAK2 fusion protein only elicits subtle effects on myeloid differentiation, and is unable to transform early hematopoietic progenitors. However, phosphoprotein and transcriptome analyses reveal that RUNX1-JAK2 constitutively activates JAK-STAT signaling in differentiating hiPSCs and at the same time upregulates MYC targets—confirming the interaction between these pathways. This proof-of-principle study indicates that conditional expression of oncogenic fusion proteins in combination with hematopoietic differentiation of hiPSCs may be applicable to leukemia-relevant disease modeling.

## 1. Introduction

B-cell precursor acute lymphoblastic leukemia (B-ALL) is the most frequent pediatric malignancy and a clinically and genetically heterogeneous disease [1,2,3,4,5]. A genetically diverse B-ALL subgroup comprises cases with rearrangements affecting genes involved in cytokine-receptor or kinase signaling, such as *ABL1*, *ABL2*, *PDGFRB*, *CSF1R*, *JAK2*, *EPOR*, and *CRLF2* [6,7]. These alterations elicit similar gene expression signatures and often confer failure to standard multidrug treatment [1]. Theoretically, at least some of these fusion proteins may constitute a dual-hit oncogenic mutation. On the one hand, constitutive kinase-activation induces proliferative and/or antiapoptotic signaling pathways. On the other hand, interference with the function of the other fusion partner—for example, a developmental transcription factor—blocks differentiation, as is the case for EBF1-PDGFRB and PAX5-JAK2 [8,9,10].

Here, we employed in vitro differentiation of genetically engineered hiPSCs toward hematopoietic progenitors as a cellular model system to study the function of JAK2 fusion proteins. Since in vitro differentiation of hiPSCs toward B-lymphoid cells remains a challenging task, we aimed to investigate a JAK2 fusion, which occurs in leukemia of the myeloid and lymphoid lineages, and whose expression is driven by an N-terminal partner at the onset of hematopoietic development. When routine diagnostics identified a pediatric B-ALL patient with an in-frame RUNX1-JAK2 fusion, which has also been proposed to be present in a case of myeloproliferative neoplasm (MPN) [11], we opted to investigate its impact on hematopoietic differentiation. Similar fusion proteins, such as PCM1-JAK2, were found in myeloid and lymphoid malignancies [12].

Both fusion partners, *RUNX1* and *JAK2*, are prominent leukemia-associated genes that are often affected by genomic rearrangements or mutations [12,13,14,15,16]. Cooperation of RUNX1 and JAK-STAT alterations has already been suggested to play a role in acute myeloid leukemia (AML) development [17,18].

The N-terminal fusion partner RUNX1 is a transcription factor, which is essential for early hematopoiesis [19]. RUNX1 is especially crucial for hematopoietic specification during endothelial to hematopoietic transformation (EHT) [20]. At later stages of development, it is involved in the differentiation, and survival of, for example, the megakaryocytic lineage [21,22,23]. In leukemia-associated genomic rearrangements, RUNX1 may either represent a C-terminal fusion partner—for example, in *ETV6-RUNX1*-positive B-ALL—or it may be N-terminally fused to proteins, such as eight-twenty-one family members (RUNX1T1, CBFA2T2, CBFA2T3) in AML [24,25,26]. RUNX1 fusions [13], as well as germline or somatic mutations [14], are associated with several different myeloid or lymphoid dysplastic or neoplastic hematological diseases.

The C-terminal fusion partner, the non-receptor tyrosine kinase JAK2, plays crucial roles in hematopoiesis, proliferation, differentiation, and survival [27]. It is involved in the signaling cascade from various cytokine receptors to downstream targets, including the signal transducers and activators of transcription (STATs). Upon binding of cytokine ligands to their respective receptors, conformational changes lead to auto-phosphorylation and activation of bound JAK2, which subsequently phosphorylates cytoplasmic STATs. Phosphorylated STATs then dimerize and shuttle to the nucleus, acting as sequence-specific transcription factors [28,29]. An important canonical JAK2 phosphorylation target protein, STAT5, is essential for the survival of normal, as well as leukemic stem and progenitor cells [29,30,31]. Aberrations of *JAK2*, either point-mutations or C-terminal fusions to *PCM1*, *ETV6*, *PAX5*, *EBF1*, or others [12], are recurrently associated with different hematopoietic malignancies and result in constitutive activation of JAK-STAT signaling [15,32]. Notably, while some JAK2 fusion proteins contain self-interaction domains that cause auto-phosphorylation and canonical downstream target phosphorylation in the cytoplasm, others lack oligomerization motifs and/or localize to the nucleus, suggesting distinct functional modes [9,33,34].

Several JAK2 fusion proteins have been demonstrated to result in constitutive activation of JAK-STAT signaling [15,32]. To prove activation of this pathway, generally, murine lymphoid Ba/F3 cells are used, which upon kinase activation acquire cytokine-independent growth and display enhanced phosphorylation of STAT1, STAT3, and STAT5 (pSTAT1/3/5) [9,35,36]. Increased pSTAT5 levels were also detected in many primary human leukemia samples [37,38]. However, neither in cell lines nor in primary leukemia cells, the impact of a fusion protein on the dynamics of hematopoietic development can be analyzed.

In contrast, directed in vitro hematopoietic differentiation of hiPSCs facilitates the modeling of blood diseases throughout development. For this purpose, differentiation phenotypes of either genetically engineered or patient-derived hiPSCs are compared to normal isogenic controls. Intriguingly, most types of leukemia appear to be refractory to reprogramming toward pluripotency [39,40]. However, a few hiPSC lines were derived from AML patients and were used to model important aspects of disease development in vitro [41,42].

Here, we have established a new well-controlled in vitro model system using genetically modified hiPSCs to determine the impact of the RUNX1-JAK2 fusion on hematopoietic differentiation and downstream pathways in an otherwise normal genomic background. Our data provide strong evidence that in differentiating hiPSCs RUNX1-JAK2 constitutively activates JAK-STAT and stimulates the MYC pathway.

## 2. Results and Discussion

### 2.1. Establishment of Knock-in hiPSC Lines Harboring a RUNX1-JAK2 Fusion

**Figure 1:**
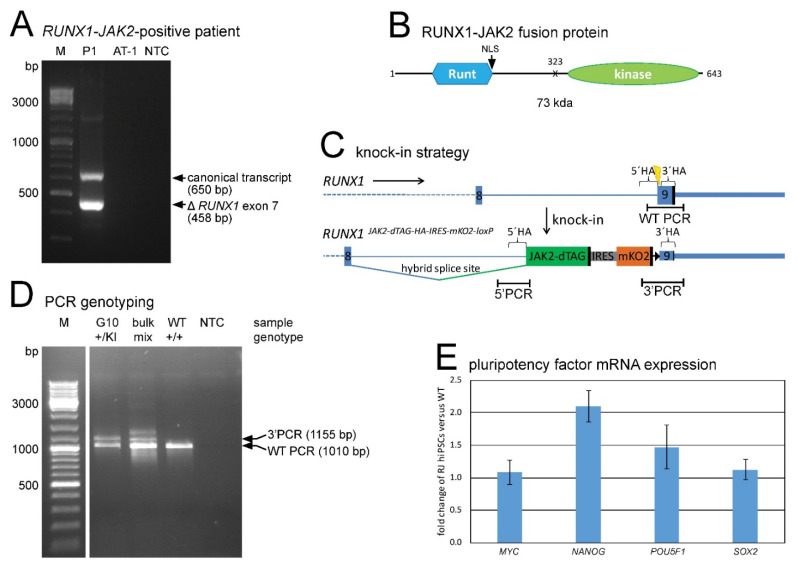
*RUNX1-JAK2* fusion gene identification and establishment of corresponding knock-in hiPSC lines. (**A**) Reverse transcription polymerase chain reaction (RT-PCR) with *RUNX1* forward and *JAK2* reverse primers showed expression of two in-frame fusion transcripts (the canonical full-length and a splice variant lacking *RUNX1* exon 7) in patient P1. The *ETV6-RUNX1*-positive cell line AT-1 and a no template control (NTC) served as negative controls (bp, base pair). (**B**) Putative RUNX1-JAK2 protein structure depicted with the Runt homology domain and the nuclear localization signal (NLS) of RUNX1 and the JH1 tyrosine kinase domain of JAK2, the breakpoint (X), the amino acid positions, and the expected molecular weight (kda, kilodalton). (**C**) Knock-in strategy: The *JAK2* encoded part fused to a dTAG, and an IRES-mKO2 cassette were inserted into *RUNX1* exon 9. Locations of CRISPR/Cas9 target site (yellow bolt), homology arms (5′HA and 3′HA), PCR amplicons (5′ and 3′ PCR detecting the knock-in flanks, and WT PCR the wild-type allele) and stop codons (black vertical bars) are shown. A loxP site (black triangle) persisted after Cre-mediated excision of the puromycin resistance cassette (not shown). (**D**) Three primer PCR of a knock-in clone example (G10) and a cell bulk yielded WT and 3′ PCR products (WT, parental cell line; NTC, no template control). Deduced genotypes are indicated (+, wild-type allele; KI, knock-in allele; mix, +/KI mixed with +/+ or +/floxed). (**E**) Expression of the four indicated pluripotency factors in *RUNX1-JAK2* hiPSCs was determined by quantitative RT-PCR and normalized to *GUSB* and *ABL1*. Fold expression of 8 different knock-in lines (RJ, n = 8; mean ± standard deviation) compared to wild-type (WT) hiPSCs is shown.

In this study, we investigated the impact of the fusion protein RUNX1-JAK2 on hematopoietic development. The chimeric transcript detected in a B-ALL patient consists of the first eight exons of *RUNX1* fused to *JAK2* exons 19–25 (Figure 1A), consequently encoding a protein that consists mainly of the RUNX1 Runt DNA-binding and JAK2 JH1 tyrosine kinase domains (Figure 1B).

We used genome editing to establish hiPSC lines carrying this so far uncharacterized, putatively leukemogenic fusion. Since we intended to express RUNX1-JAK2 at physiological levels via relevant regulatory elements, we inserted the *JAK2* encoding fusion part into one endogenous *RUNX1* allele in such a way that a hybrid splice site, consisting of *RUNX1* intronic and *JAK2* exonic sequences, was created (Figure 1C). Thus, the resulting *RUNX1-JAK2* knock-in allele mimics the fusion gene present in the patient. We also added a C-terminal dTAG degron and a tandem hemagglutinin (HA) epitope tag to facilitate fusion protein depletion [43] and detection, respectively. Furthermore, a downstream internal ribosome entry site monomeric Kusabira Orange 2 (IRES-mKO2) reporter cassette was included to monitor *RUNX1* expression in live cells. Finally, a floxed puromycin resistance cassette was temporarily inserted to allow for the selection of successfully genome-edited cells, but was later excised by Cre-mediated recombination to prevent unintended interferences.

To avoid the formation of insertions or deletions in the second *RUNX1* allele, as well as at off-target sites, due to error-prone DNA double-strand break repair, we employed an *in trans* paired nicking approach [44,45] rather than conventional Clustered Regularly Interspaced Short Palindromic Repeats (CRISPR) mediated knock-in. We used a CRISPR/Cas9-D10A ribonucleoprotein complex targeting *RUNX1* and a donor template vector with the same *RUNX1* nuclease target site flanking both homology arms. Eight hiPSC clones with transgene insertion in one *RUNX1* locus and one unaltered wild-type allele were established (Figure 1D, Supplementary Figures S1A and S2). According to the informative heterozygous single nucleotide polymorphism (SNP) rs13051066, the insertion took place to the same extent on both *RUNX1* alleles (4 clones each), but none of the clones harbored a rarely occurring biallelic insertion. Based on SNP array analysis, no copy number alterations were detectable (data not shown), suggesting that no other gross genomic rearrangements had occurred. Single cell cloning during reprogramming or genetic engineering often goes along with unintended selection for *TP53* mutations, because these confer growth and survival advantage [46,47]. Such confounding alterations were excluded by RNA-seq data analysis (Section 2.6). All hiPSC knock-in cell lines, when cultured under hypoxia in TeSR media (supplemented with ROCK inhibitor Y-27632 during splitting, transfecting, freezing, and thawing) on Matrigel coating, expressed high levels of pluripotency marker mRNA (Figure 1E) and protein (Supplementary Figure S1B), while neither wild-type RUNX1 nor RUNX1-JAK2 nor mKO2 protein were yet expressed (data not shown).

### 2.2. RUNX1-JAK2 Fusion Protein Expression upon Hematopoietic Differentiation

**Figure 2:**
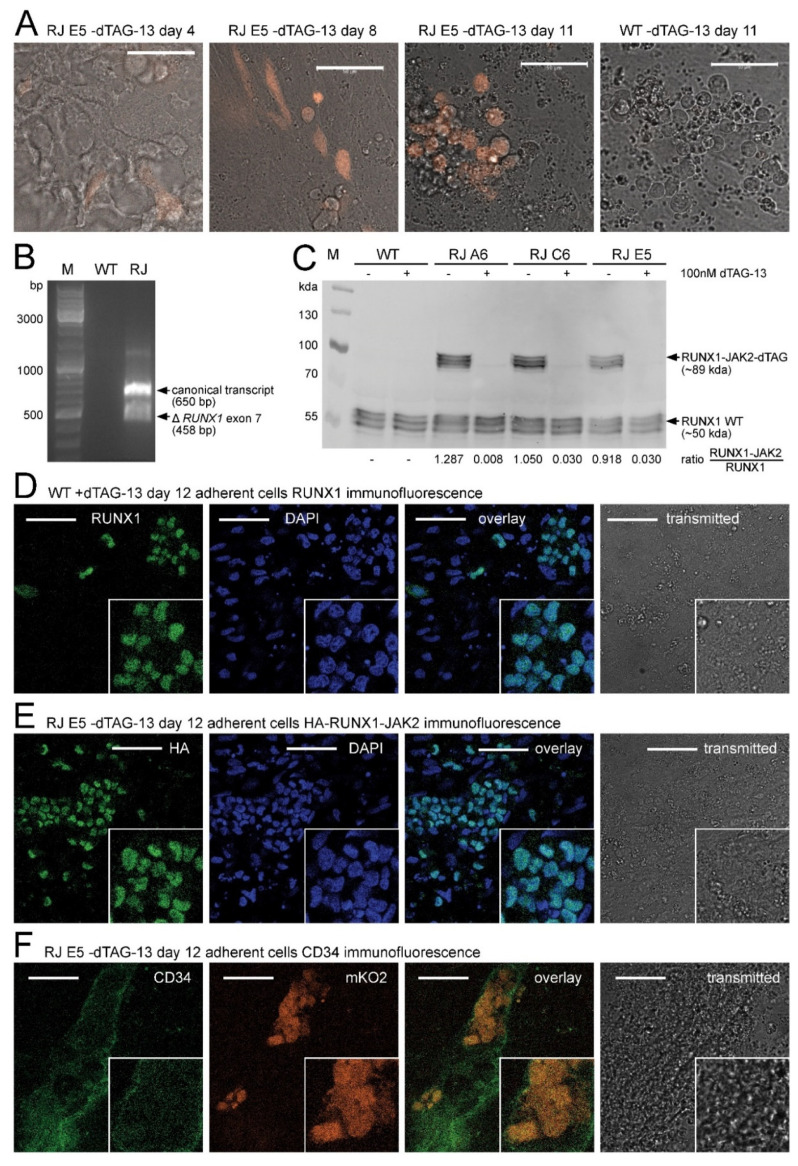
RUNX1-JAK2 expression during hematopoietic differentiation. (**A**) Representative live cell images (orange mKO2 fluorescence and grey transmitted light overlay) during hematopoietic differentiation. *RUNX1-JAK2* knock-in (RJ) and wild-type (WT) cells at indicated timepoint are shown. (**B**) RT-PCR revealed correct expression of two *RUNX1-JAK2* transcript variants in differentiated RJ, but not in WT cells. (**C**) RUNX1 and RUNX1-JAK2 fusion protein expression in differentiated dTAG-13-treated or untreated WT and three RJ lines (unstarved adherent cell lysates) was analyzed by Western blot with an RUNX1-specific antibody. Protein variants of slightly different molecular weights are expressed due to usage of the proximal and distal promoters, alternative splicing (e.g., *RUNX1* exon 7 skipping), and posttranslational modifications. Signal ratios of RUNX1-JAK2 fusion to wild-type RUNX1 proteins are indicated below. (**D**–**F**) Immunofluorescence stainings were performed with unstarved adherent cells fixed after 12 days of differentiation. (**D**) RUNX1 protein localization (green) in wild-type cells was determined by indirect immunofluorescence; DAPI counterstain (blue), overlay, and transmitted light (grey) pictures are also shown. (**E**) Untreated RJ clone E5 cells were stained for HA-tagged RUNX1-JAK2 protein (green) as in D. (**F**) Concomitant expression of CD34 (APC fluorescence in green) and mKO2 (orange) was detected in untreated RJ E5 cells by direct immunofluorescence. White bars correspond to 50 µm; M, molecular weight markers; bp, base pair; kda, kilodalton; close-ups (twofold magnification) are shown on the bottom right of immunofluorescence pictures.

While inactive in the pluripotent state, after four days of directed hematopoietic differentiation [48], a fraction of the cells started to express the mKO2 reporter from the *RUNX1* locus. From day 8 onwards, numerous mKO2-expressing cells performed EHT similar to wild-type lines and remained mKO2-positive as suspension cells (Figure 2A). Comparable to patient P1 (Figure 1A), the *RUNX1-JAK2* fusion gene was transcribed from the distal and proximal promoters and correctly spliced (Figure 2B).

The fusion protein of expected size was translated at a level very similar to that of RUNX1 (Figure 2C). Like the wild-type protein (Figure 2D, Supplementary Figure S3A–C), also RUNX1-JAK2 localized to the cell nucleus as demonstrated by HA-tag immunofluorescence (Figure 2E, Supplementary Figure S3D,E).

Moreover, at this stage of differentiation, the mKO2-positive cells exhibited CD34 surface expression (Figure 2F), suggesting activation of the *RUNX1* wild-type and *RUNX1-JAK2* knock-in alleles primarily in CD34+ CD43+ and CD34+ CD144+ hemato-endothelial progenitors (Supplementary Figures S4 and S5). Upon treatment with 100 nM dTAG-13 compound throughout differentiation, the RUNX1-JAK2 protein was continuously degraded, and its expression level was substantially reduced (Figure 2C). Consequently, our well-controlled, conditional expression approach enabled us to investigate the impact of RUNX1-JAK2 on hematopoietic differentiation.

### 2.3. RUNX1 Haploinsufficiency of RUNX1-JAK2 Knock-In Cell Lines

**Figure 3:**
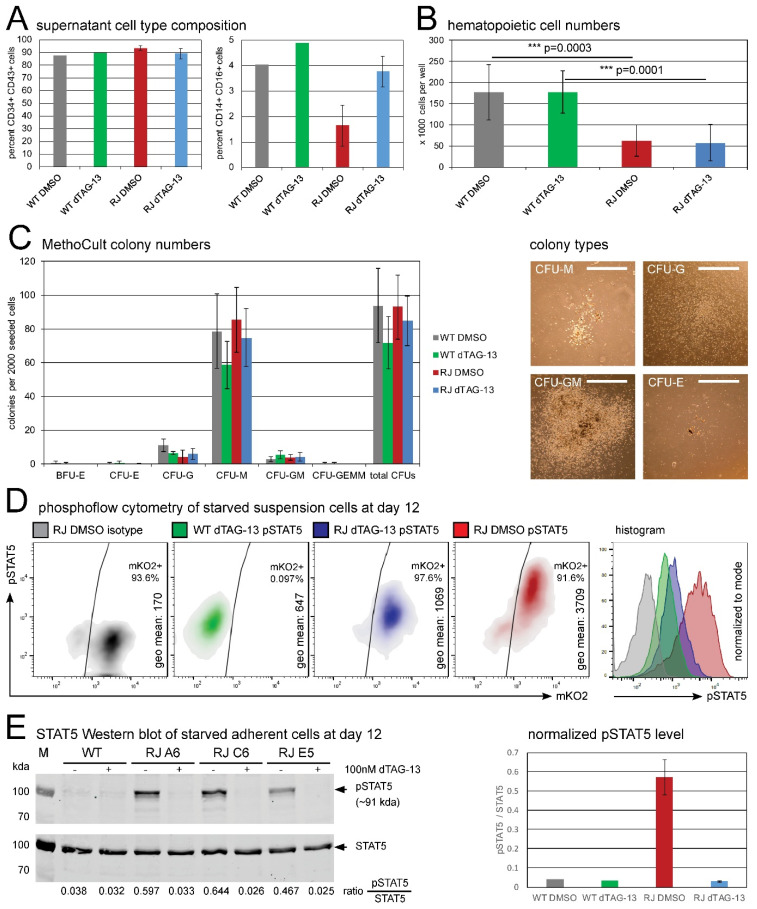
Functional analyses of *RUNX1-JAK2* and wild-type hematopoietic progenitor cells. (**A**) Supernatant cells were harvested from day 12 differentiation cultures, and flow cytometry for surface markers CD34, CD43, CD14, and CD16 was performed. Mean percentages for CD34+ CD43+ hematopoietic progenitors (left) and CD14+ CD16+ monocytes (left) are shown (wild-type, WT, n = 1; *RUNX1-JAK2*, RJ, n = 3; dTAG-13 or DMSO-treated cells; error bars represent standard deviations). (**B**) Total live suspension cells were harvested and counted (wild-type, WT, n = 5; *RUNX1-JAK2*, RJ, n = 13; 8 different clones; dTAG-13-treated or DMSO controls). Mean cell numbers ± standard deviation per well of a 12-well plate and adjusted *p*-values of the indicated comparisons are shown (analysis of variance, ANOVA; ***, very high significance). (**C**) MethoCult assays: 2000 supernatant cells were seeded per well of a 6-well plate in methylcellulose medium containing cytokines, and after 14 days of culture, the numbers and types of colonies were enumerated (left; WT, n = 3; RJ, n = 7, 7 different clones; BFU, burst forming unit; CFU, colony forming unit; E, erythrocyte; G, granulocyte; M, macrophage; GM, granulocyte and macrophage; GEMM, granulocyte, erythrocyte, macrophage, and megakaryocyte; means ± standard deviation). Representative pictures of different CFU types are also shown (right; white bars correspond to 1 mm). (**D**) Supernatant cells were starved, fixed, permeabilized, stained with an antibody specific for phosphorylated STAT5, and analyzed by flow cytometry. Density and histogram plots of intact single cells (gated according to forward and sideward scatter), pSTAT5 geometrical means, and percentages of mKO2 positivity are depicted for untreated RJ clone E5 cells (RJ DMSO, red) and controls (RJ dTAG-13, blue; WT dTAG-13, green; isotype IgG, grey). One representative result of four independent experiments is shown. (**E**) Western blot analysis of dTAG-13-treated or untreated WT and three clonal RJ lines (lysates of differentiated adherent cells starved for 5 h in unsupplemented IMDM) was performed for pSTAT5 and total STAT5 protein (left; kda, kilodalton). Signal ratios of phosphorylated to total STAT5 protein are presented below and summarized in a bar chart (right; means ± standard deviation). Percentages of the obtained RUNX1-JAK2-positive hemato-endothelial cells in the differentiation cultures and consequently pSTAT5 signal levels were variable.

To analyze the effects of RUNX1-JAK2 expression on early hematopoiesis, we first harvested and counted live hematopoietic cells floating in the supernatant after 12 days of hiPSC differentiation [48]. This cell population was highly enriched in hematopoietic progenitors (on average 90% CD34+ CD43+ double positive cells; Figure 3A, Supplementary Figure S6; [49]). While the percentage of progenitors was slightly increased in RUNX1-JAK2-expressing lines, a monocytic commitment was decreased about twofold (Figure 3A, Supplementary Figure S7). Notably, the observed suspension cell yield was significantly diminished (about threefold) in the knock-in cell lines compared to wild-type controls (Figure 3B). Since this observation remained valid regardless of whether the fusion was expressed or degraded (i.e., without or upon dTAG-13 treatment, respectively; Figure 3B), this defect is most probably due to *RUNX1* wild-type haploinsufficiency resulting from the insertion of the fusion partner into one allele. Hematopoietic deficits arising from impaired EHT have already been described for *RUNX1* knock-out mice [19,20], while RUNX1 overexpression has been shown to enhance hematopoietic output in vitro [50]. Together, these findings align with the current understanding that *RUNX1* gene dosage plays a critical role in developmental hematopoiesis [21,22].

Our current model correctly accounts for the loss of one wild-type *RUNX1* allele present in leukemia, but not for the concomitant *JAK2* hemizygosity. However, since JAK2 activity is primarily controlled by phosphorylation and inhibition of the protein [27,28], and since no significantly altered phenotype was observed in heterozygous *Jak2* knock-out mice [51], we assume that the effects of its haploinsufficiency are negligible.

### 2.4. Clonogenic Potential of RUNX1-JAK2-Expressing Hematopoietic Progenitors

Next, we performed cytokine-enriched methylcellulose assays to investigate the clonogenic potential of hematopoietic stem and progenitor cells (HSPCs) present in the supernatants of day 12 differentiation cultures. We did not observe significant differences in colony forming unit (CFU) numbers, types, or sizes between parental and isogenic *RUNX1-JAK2* lines (Figure 3C). Despite the previously observed reduced hematopoietic cell yield from *RUNX1-JAK2* hiPSC lines (Figure 3B), the composition (Figure 3A, Supplementary Figures S6 and S7) and clonogenic quality of the HSPCs (Figure 3C) was not evidently altered, indicating that *RUNX1* haploinsufficiency affects mainly EHT, but not subsequent hematopoietic lineage differentiation surveyed in the cytokine-enriched methylcellulose assay [20].

Notably, at least in our experimental setting, the expression of RUNX1-JAK2 fusion protein did not cause any obvious differences regarding clonogenicity (Figure 3C). Like parental wild-type and dTAG-13-treated knock-in progenitors, also RUNX1-JAK2-expressing cells formed only a few very small CFU-G or CFU-M colonies in methylcellulose without cytokines (data not shown), suggesting that cytokine-dependency still prevailed. Moreover, differentiated cells harvested from primary methylcellulose cultures were in general unable to form secondary colonies (data not shown). In contrast, transposon-mediated expression of RUNX1-JAK2 protein resulted in IL3-independent growth of Ba/F3 cells in liquid culture (Supplementary Figure S8A).

These results imply that RUNX1-JAK2 expression *per se* does not lead to oncogenic transformation of early hematopoietic progenitors derived from hiPSCs under the experimental conditions used. However, we cannot rule out that cells at other stages of development, such as early B-cell progenitors, whose efficient generation by directed in vitro differentiation of hiPSCs remains highly challenging [52,53], are susceptible to transformation by RUNX1-JAK2 [31]. Noteworthy, compared to native in vivo generated hematopoietic stem cells, HSPCs derived from hiPSCs have distinct properties and are, so far, incapable of long-term engraftment in mice, unless an adequate set of multiple transcription factors with oncogenic properties is ectopically expressed [54,55,56]. Hence, it is still possible that native HSPCs are more permissive to RUNX1-JAK2-mediated transformation.

### 2.5. RUNX1-JAK2 Constitutively Activates STAT5 in Differentiated hiPSCs

In the next step, we addressed the question of whether the expression of RUNX1-JAK2 leads to constitutive activation of the JAK-STAT pathway in hiPSC-derived progenitors as it does in Ba/F3 cells (Supplementary Figure S8B,C). For this purpose, we harvested hematopoietic cells from differentiation culture supernatants and performed short-term starvation in a medium without supplements to reduce steady state signaling to basal levels. Then, we conducted phosphoflow cytometric analysis [57] of pSTAT5, which is activated by JAK2-mediated phosphorylation. As shown in Figure 3D, despite prior starvation, RUNX1-JAK2-expressing suspension cells displayed high pSTAT5 levels, whereas those of wild-type cells were much lower. STAT5 phosphorylation was only slightly higher in dTAG-13-treated *RUNX1-JAK2* than in wild-type cells, supposedly due to the presence of residual, not yet degraded fusion protein. Western blot analysis also showed an approximately 18-fold increase of STAT5 phosphorylation in starved differentiated adherent RUNX1-JAK2-expressing cells (Figure 3E), of which, in fact, only a variable fraction expressed the fusion protein.

Furthermore, we investigated the phosphorylation of STAT1 and STAT3. In Ba/F3 cells, pSTAT1 and pSTAT3 levels were highly increased by RUNX1-JAK2 (Supplementary Figure S9A,B). In contrast, in the adherent fraction of hiPSCs differentiated for 12 days, despite several hours of starvation, pSTAT1 and pSTAT3 levels remained high, and no obvious further induction by RUNX1-JAK2 was detectable (Supplementary Figure S9C,D). This finding is supposedly due to the self-stimulation of endothelial and mesenchymal cells via pathways, such as PDGF, FGF, and VEGF. Thus, the differences in RUNX1-JAK2-mediated STAT1 and STAT3 phosphorylation between Ba/F3 and adherent differentiated hiPSCs may well be cellular context-dependent. However, in both instances, exogenous stimulation by cytokines appears to be required for STAT5 phosphorylation unless complemented by a kinase-activating fusion protein. These data imply that the lack of transforming capacity of RUNX1-JAK2 in hiPSC-derived progenitors is not due to its general failure to activate JAK-STAT signaling.

### 2.6. Impact of RUNX1-JAK2 on the Hematopoietic Transcriptional Landscape

Finally, to explore the impact of the RUNX1-JAK2 fusion protein on genome-wide transcription, we performed RNA-seq of hematopoietic cell bulks after 12 days of differentiation and 4 h of starvation. Three independent experiments were conducted with altogether three wild-type controls (WT) and six *RUNX1-JAK2* clones (RJ) as biological replicates, each either dTAG-13-treated or not.

**Figure 4:**
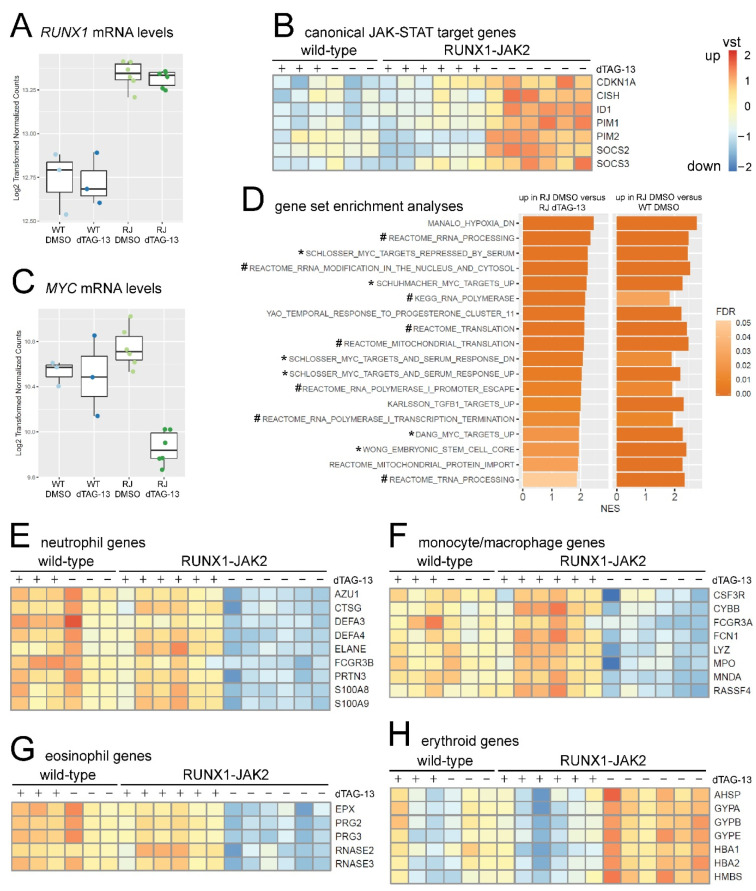
Gene expression analyses of starved RUNX1-JAK2-expressing hematopoietic cells. RNA-seq was conducted with wild-type (WT, n = 3) and *RUNX1-JAK2* (RJ, n = 6) cells differentiated for 12 days either with (+, dTAG-13) or without (−, DMSO) degrader treatment. (**A**) Boxplot showing normalized log_2_-transformed *RUNX1* mRNA expression levels. (**B**) Expression profiles of canonical pSTAT5 targets. Heat-maps show batch-corrected and variance-stabilization-transformed (vst) log_2_-fold changes from low (blue) to high expression (orange). (**C**) Boxplot displaying normalized log_2_-transformed *MYC* mRNA expression levels. (**D**) Gene sets significantly upregulated with false discovery rate (FDR) q-values ≤ 0.05 in both enrichment analyses, untreated RJ versus dTAG-13-treated RJ and untreated RJ versus untreated WT, are listed. The *x*-axis represents normalized enrichment scores (NES), the brightness of the bar FDR values, gene sets directly related to MYC and RNA biology are marked with asterisks (*) and hashes (#), respectively. Expression profiles of selected significantly regulated genes related to neutrophils (**E**), monocytes and macrophages (**F**), eosinophils (**G**), and erythrocytes (**H**) are depicted.

Based on the rs13051066 SNP frequencies in the RNA-seq reads, the *RUNX1* and *RUNX1-JAK2* knock-in alleles were transcribed at similar rates in the differentiated transgenic lines. Remarkably, according to differential gene expression analysis, the combined mRNA level of *RUNX1* and *RUNX1-JAK2* was elevated by roughly 50% (Figure 4A). Consequently, wild-type *RUNX1* mRNA reached on average three quarters of the parental cell level. We assume that about a third of the heterozygous progenitors stochastically expressed *RUNX1* at levels sufficient for EHT, thus these cells were able to transit into the supernatant and were subsequently harvested from the cultures (Figure 3A,B).

Nonetheless, the reduced expression of megakaryocyte/thrombocyte associated transcripts in untreated, as well as dTAG-13-treated *RUNX1-JAK2* cells (Supplementary Figure S10A), indicates a mild impairment of megakaryocytic differentiation due to *RUNX1* haploinsufficiency [23].

Furthermore, two unrelated coding (*CLIC6*, *RCAN1*; Supplementary Figure S10B,C) and two non-coding loci (*LINC01426*, *LINC00160*) immediately downstream of *RUNX1* were slightly upregulated, supposedly due to weak *in cis* activation by the insert. As expected, *JAK2* and *FKBP1A,* because of the additionally inserted and expressed 3′ fusion partner and dTAG sequences, also exhibited slightly increased total transcript levels (Supplementary Figure S10D,E).

Next, we performed differential gene expression and gene set enrichment analysis (GSEA) using pre-ranked shrunken log_2_-fold change lists. Four-hundred-and-seventeen genes were differentially regulated by RUNX1-JAK2 expression (83 up- and 334 down-regulated genes in untreated RJ versus dTAG-13-treated RJ; change ≥ 2-fold and adjusted *p*-value ≤ 0.01). In contrast, dTAG-13 treatment had no significant influence on gene expression of WT cells (Supplementary Table S1). RUNX1-JAK2-expressing cells, as expected for constitutive JAK-STAT signaling, significantly upregulated canonical pSTAT5 target genes (Figure 4B), confirming cytokine-independent transcriptional activation of signaling mediators further downstream. In this context, it is important to note that several of these targets are involved in negative feedback circuits by either attenuating JAK-STAT signaling (e.g., SOCS2, [27]) or curtailing excessive proliferation (e.g., CDKN1A, [58]). This might explain why GSEA did not reach high significance levels for larger JAK-STAT-related gene sets (Supplementary Table S1).

Intriguingly, although transcript levels of the proto-oncogenes *MYC* (Figure 4C) and *MYCN* (Supplementary Figure S10F) were only marginally elevated, and MYC protein was not considerably altered by RUNX1-JAK2 expression (Supplementary Figure S10G), various MYC transcriptional target gene sets were significantly upregulated (Figure 4D). This suggests high MYC activity, which may occur at the level of posttranslational modifications and co-activating or repressing interaction partners [59,60]. Although the exact underlying mechanism remains elusive, our observation affirms the crosstalk between the JAK-STAT and MYC pathways as already described for NK-cell leukemia [61] and B-ALL [37,62]. This finding also offers an explanation for the concomitant upregulation of genes involved in RNA transcription, processing and translation (Figure 4D), which is consistent with MYC-mediated transcriptional amplification [63]. In addition, RUNX1-JAK2 appears to inversely regulate hypoxia-related gene sets (Figure 4D, Supplementary Table S1), which is again in line with downregulation of MYC targets mediated by HIF1A at low oxygen tension [64].

Moreover, while RUNX1-JAK2-expressing cells showed significantly decreased levels of monocyte and granulocyte-related myeloid markers (Figure 4E–G; consistent with Figure 3A and Supplementary Figure S7), they displayed increased transcription of erythroid genes, such as the α-globin and glycophorin loci (Figure 4H). This suggests that the fusion protein promotes at least a minor differentiation bias toward the erythroid at the expense of other myeloid cell lineages as already described for other STAT5 hyperactivation models [65,66]. Consequently, multiple gene sets related to macrophages or granulocytes were underrepresented in RUNX1-JAK2-expressing cells, such as targets of interferon beta 1 inflammatory signaling (Supplementary Figure S11A).

However, in cytokine-enriched methylcellulose assays, these lineage-specific transcriptional changes are supposedly too subtle to elicit significant changes in CFU development (Figure 3C), or, as outlined above, they might be due to inherent differences in the properties of hematopoietic progenitors derived from hiPSCs.

We also did not observe increased clonogenicity or enhanced hematopoietic differentiation upon RUNX1-JAK2 expression, as described by others for STAT5A-hyperactivated human cord blood HSPCs or murine embryonic stem cells, respectively [66,67]. Possible explanations for these divergences are differences in cell type, culture conditions, the intensity of STAT5 activation [65], or the concomitant *RUNX1* haploinsufficiency. Likewise, in the corresponding human leukemia *RUNX1* is hemizygous, however, the *RUNX1-JAK2*-causing translocation supposedly took place at a more advanced developmental stage. Furthermore, the lack of significantly deregulated gene sets related to RUNX transcription factors (GSEA of dTAG-13-treated RJ versus WT, Supplementary Table S1) suggests that RUNX1 function is largely intact also because it may be partially compensated by selection for cells with increased mRNA levels (Figure 4A). Hence, the observed transcriptional changes appear to be governed primarily by RUNX1-JAK2-mediated JAK-STAT signaling and subsequent activation of the MYC pathway. Although other studies proposed direct MYC upregulation on the mRNA or protein level by JAK-STAT signaling, our data hint at an alternative positive interaction between the two pathways either by protein activity regulation or by co-activation of target loci. The exact mechanisms underlying this synergy remain to be addressed in future research.

## 3. Materials and Methods

### 3.1. Reverse Transcription, PCR, and RNA-seq

Patient P1 was enrolled in the ALL-BFM 2009 (NCT01117441) clinical trial. Total RNA from diagnostic bone marrow was extracted using the QIAamp RNA Blood Mini kit (Qiagen, Hilden, Germany). The B-ALL cell line AT-1 was used as a negative control [68]. The expression of the *RUNX1-JAK2* fusion gene was confirmed by RT-PCR with High Capacity cDNA Reverse Transcription Kit (Thermo Scientific), HotStarTaq (Qiagen), and 500 nM primers RUNX1-ex5-F3 and JAK2-ex20-R1, followed by Sanger-sequencing. Sequence analysis was performed with CLC Workbench 7.9.1 (Qiagen). The fusion transcript consisted of *RUNX1* exons 1–8 (Ensembl transcript ENST00000437180.5) and *JAK2* exons 19–25 (ENST00000381652.4). For *RUNX1*, an alternatively spliced in-frame transcript variant lacking exon 7 has been described (ENST00000399240.5).

Total RNA of hiPSCs and differentiated derivative cells was extracted using TRIzol reagent (Thermo Fisher Scientific) following the manufacturer’s protocol with glycogen as co-precipitant. Complementary DNA (cDNA) was synthesized from 2 µg total RNA using 500 ng of each random and oligo-dT_18_ primers and M-MLV reverse transcriptase (Promega). Correct splicing of the knock-in fusion transcript was verified by RT-PCR with HotStarTaq (Qiagen), and 500 nM primers RUNX1-ex5-F3 and JAK2-ex20-R1, and Sanger-sequencing. RT-qPCRs for *POU5F1*, *MYC*, *SOX2*, *NANOG*, *GUSB*, and *ABL1* were conducted in triplicates on a 7500-Fast cycler (Applied Biosystems, Waltham, MA, USA) with cDNA corresponding to 40 ng total RNA per 20 µL reaction using 200 nM forward and reverse primers and iTaq Universal SYBR-green Supermix (Bio-Rad). PCR efficiencies of 90–100% were verified by standard dilution series and specificity by melt curve analyses. Relative quantification was performed by normalization to ROX reference dye, *GUSB* and *ABL1* housekeeping gene expression, and parental hiPSCs using the 2^−ΔΔCt^ method. All primers are listed in Supplementary Table S2.

Library preparation and RNA-seq were conducted at the Next Generation Sequencing Facility of the Vienna BioCenter Core Facilities Austria (VBCF; https://www.viennabiocenter.org/vbcf/next-generation-sequencing/, accessed on 8 July 2021). In brief, 500 ng total RNA was enriched for polyA-containing mRNAs and converted to barcoded libraries using the NEBNext Ultra II kit (New England Biolabs). Eighteen samples were multiplexed and single end 100 bp reads sequenced on a NovaSeq 6000 SP XP flow cell. Demultiplexed reads were mapped to human genome GRCh38 without alt loci (ftp://ftp.ncbi.nlm.nih.gov/genomes/all/GCA/000/001/405/GCA_000001405.15_GRCh38/seqs_for_alignment_pipelines.ucsc_ids/GCA_000001405.15_GRCh38_no_alt_analysis_set.fna.gz, accessed on 26 June 2018) using STAR 2.7.0b [69]. Further analysis was performed in R (version 3.4.4) statistical environment using Bioconductor packages [70]. Count statistics for Refseq genes were obtained by the “featureCounts” function (package Rsubread_1.28.1) using Ensembl annotation Homo_sapiens.GRCh38.100 [71]. Gene expression was normalized, batch-corrected, and analyzed using DESeq2 version 1.18.1 [72], including independent filtering with alpha = 0.05. Gene set enrichment analysis (GSEA) was performed with pre-ranked lists according to shrunken log_2_-fold changes [73] using GSEA 2.2.4 and MSigDB 7.1 subset c2 [74]. To avoid the effects of potentially confounding *RUNX1* haploinsufficiency, we only considered gene sets relevant if they were regulated significantly and equally in both comparisons, untreated RUNX1-JAK2 (RJ) versus untreated wild-type (WT), as well as untreated RJ versus dTAG-13-treated RJ. Detailed RNA-seq results are summarized in Supplementary Table S1.

### 3.2. HiPSC Culture and Differentiation

The parental episomally reprogrammed hiPSC line was purchased from Thermo Fisher Scientific (Gibco A18945; https://hpscreg.eu/cell-line/TMOi001-A, accessed on 10 December 2020). hiPSCs were routinely cultured under hypoxic conditions (37 °C, 3% O_2_, 5% CO_2_) on plates coated with hESC-qualified Matrigel (Corning) in mTeSR1, mTeSR-Plus or TeSR-E8 medium (all from STEMCELL Technologies). Cells were passaged every 3–4 days at a split ratio of about 1:6 using StemPro Accutase (Thermo Fisher Scientific, Waltham, MA, USA); 10 µM Rho-associated coiled-coil-containing protein kinase inhibitor Y-27632 (ROCK inhibitor; STEMCELL Technologies, Vancouver, BC, Canada) was added for splitting, transfecting, freezing, and thawing. Mycoplasm contamination was excluded by regular testing using a luminescent detection kit (Lonza MycoAlert).

The STEMdiff Hematopoietic Kit (STEMCELL Technologies) was used for differentiation according to the manufacturer’s protocol (Document #29768 v1_2_0), albeit with minor changes [48,54]. Briefly, 2000 hiPSCs were seeded as clumps per well of a Matrigel-coated 12-well plate. Differentiation was started 4 days later by normoxic cell culture with STEMdiff hematopoietic differentiation medium and supplement A (containing BMP4, FGF2, VEGFA) for the first day with 3 µM CHIR99021 glycogen synthase kinase 3 inhibitor (Sigma-Aldrich, St. Louis, MO, USA) and another 2 days without. Next, cells were cultured for another 9 days in a differentiation medium with supplement B (containing BMP4, FGF2, VEGFA, SCF, FLT3L, TPO) with half media changes every 2–3 days. DMSO vehicle control (140 µM) only or 100 nM dTAG-13 compound (kindly provided by Nathanael Gray, Dana-Farber Cancer Institute, Boston, MA, USA) was also present from differentiation day 2 onwards. Suspended and loosely attached cells were harvested on day 12 for clonogenicity assays, flow cytometry, and RNA-seq, while the remaining adherent cell fraction was used for RT-PCR, Western blotting, and immunofluorescence.

### 3.3. Genetic Engineering and Generation of Single Cell Clones

In trans paired nicking [44] was employed to genetically modify one, but not the other *RUNX1* allele at the intron 8 to exon 9 junction and to insert the respective *JAK2* exons. The pUC57-simple backbone donor vector contained, flanked by *RUNX1* CRISPR target sites and homology arms of 626 bp for the 5′ (5′HA) and 475 bp for the 3′ end (3′HA), an insert consisting of the *JAK2* coding fusion part (spanning exons 19–25) in frame with a (GGGGS)_3_ linker, an FKBP1A-F36V-degron (dTAG; mutated ENST00000400137.9) and a tandem HA-tag. Further downstream, it contained an IRES-mKO2 and a floxed puromycin resistance expression cassette with the promoter and 3′ untranslated region (UTR) from murine *Pgk1* (PuroR). The co-transfected CRISPR/Cas9 ribonucleoprotein complex (RNP) consisted of Alt-R crRNA, tracrRNA, and Cas9/D10A nickase V3 recombinant protein (all from IDT). The selected guide RNA targeting the protospacer sequence 5′-TCAGGTCGGGTGCCGCTGCA-3′ exhibited at least three mismatches to putative off-targets in hg38 (https://wge.stemcell.sanger.ac.uk/crispr/1178695897, accessed on 10 April 2017, [75]), and high on-target efficiency was predicted by two different algorithms [76,77].

One million hiPSCs were electroporated using an Amaxa Nucleofector 2b with program A-023 and Human Stem Cell Nucleofector Kit 2 (Lonza), 5 µg circular donor plasmid and 250 pmol RNP, or, after two phases of 1-day 0.5 µg/mL puromycin (Sigma-Aldrich) selection and 2-day recovery, 5 µg pCaGGS-Cre excision vector. Following the expansion of the surviving cells, 2000 singularized cells were seeded into TESR-E8 containing 10% CloneR supplement (STEMCELL technologies) on a 10-cm dish coated with Synthemax II-SC (0.025 mg/ml in 12 mL water; Corning, Corning, NY, USA). Ninety-six of the emerged colonies were picked manually, expanded, and genotyped. The recombined delta knock-in allele (sequence in Supplementary Figure S2) was not detectable in 60 clones, 23 were positive, but mixed with wild-type or floxed (still PuroR containing) knock-in cells, and 5 did not grow on the replicate plate. The remaining 8 clones were expandable and purely heterozygous for the correct insertion in one without any signs of alteration of the second *RUNX1* allele and served as biological replicates in further experiments. Cre recombinase [78], dTAG [79], and Sleeping beauty transposon [80] vectors were kind gifts from Meinrad Busslinger (IMP, Vienna, Austria), Georg Winter (CeMM, Vienna, Austria), and Rolf Marschalek (Goethe University, Frankfurt, Germany), respectively.

### 3.4. Genotyping

Genotyping PCRs for individual clones were performed using 500 nM specific primers, approximately 100 ng genomic DNA, and HotStarTaq DNA polymerase (Qiagen). DNA was isolated first by crude cell lysis in genotyping buffer (10 mM Tris-HCl pH 8.5, 50 mM KCl, 2 mM MgCl_2_, 0.45% Tween 20, 0.45% Nonidet P40 substitute, 1 mg/mL Proteinase K; all from Sigma-Aldrich) and later after clone expansion with the QIAamp DNA Blood Mini Kit (Qiagen). For the 5′ flanking PCR, primers RUNX1-in8-F5 and JAK2-ex19-R1, for the 3-primer PCR at the 3′ flanking region, primers mKO2-mid-F1, RUNX1-in8-F3, and RUNX1-ex9-R3, and for the 3′ floxed PCR primers PuroRmidF2 and RUNX1-ex9-R3 were used (oligonucleotide sequences are listed in Supplementary Table S2). PCR products (965 bp for the 5′, 1155 bp for the 3′ flanking region of the recombined, 1535 bp for the floxed knock-in allele, and 1010 bp for the wild-type allele) were purified using the Monarch kit (New England Biolabs, Ipswich, MA, USA) and sequenced with the respective forward primer (Microsynth, Switzerland). Sanger-sequences were aligned to the knock-in and wild-type alleles, respectively. The heterozygous single nucleotide polymorphism (SNP) rs13051066 slightly downstream of the 3′HA allowed the assessment of the knock-in and wild-type haplotypes, respectively.

### 3.5. Magnetic Cell Separation and Colony Forming Unit Assays

Supernatants of hematopoietic differentiation cultures were harvested on day 12, filtered through a 70 µm strainer, and live cells were purified using the MACS dead cell removal kit (Miltenyi). After Trypanblue exclusion cell counting in a Bürker-Türk chamber, 2000 cells were seeded per well of a 6-well plate into 300 µL Iscove’s Modified Dulbecco’s Medium (IMDM) containing 2% fetal bovine serum (both from Thermo Scientific) and 3 mL MethoCult semisolid medium either containing an enriched cytokine cocktail or none at all (STEMCELL Technologies H4435 or H4230, respectively). Colonies were enumerated 12 to 14 days later in a 3D microscope under dark field illumination. Live cell yields per well of 12-well plates were compared using unpaired one-way ANOVA with Tukey *post hoc* test assuming Gaussian distribution and equality of variances (GraphPad Prism 8). Colony pictures were acquired with an EVOS XL core microscope and 4× phase contrast objective (Thermo Scientific).

### 3.6. Western Blotting

Adherent cells were washed with DPBS (Dulbecco’s phosphate buffer saline) and lyzed in high salt buffer (20mM Tris-HCl pH 7.5, 400 mM NaCl, 0.5% NP-40, 0.3% Triton X-100, 0.2 mM phenylmethylsulfonyl fluoride, 1 µg/mL each of Aprotinin, Leupeptin and Pepstatin A). Cleared lysates and PageRuler prestained ladder (Thermo Scientific) were subjected to SDS-PAGE (8% acrylamide) using Tris/Glycine buffer. Tank-blotted membranes (GE Amersham Protran 0.45µm NC) were stained with Ponceau S (Sigma-Aldrich) to check equal loading (Supplementary Figures S10G, S11B and S12), incubated with blocking reagent (Roche), primary and secondary antibodies labeled with DyLight 800 or 650, and scanned on a Licor Odyssey. Local background subtracted band signal intensities were quantified using Image Studio Lite 5.2.5 (Licor). Antibodies are listed in Supplementary Table S2.

### 3.7. Microscopy and Immunofluorescence

For immunofluorescence, differentiation was performed in Matrigel coated 24-well µ-plates (Ibidi). Cells were fixed with 1% methanol-free formaldehyde in DPBS for 10 min at room temperature (RT), and for intracellular staining, cells were permeabilized with 0.2% Triton X-100 in DPBS and sequentially incubated with HA or RUNX1 (1:500), or NANOG, OCT4, or SOX2 antibody (1:50) in 2% bovine serum albumin (BSA; Sigma-Aldrich) and 0.2% Triton X-100 in DPBS, goat antimouse-IgG-AlexaFluor-488 antibody (1:2000) and 2 µg/mL 4′,6-Diamidin-2-phenylindol (DAPI; Sigma-Aldrich). One drop of mounting solution containing 10% Mowiol 4-88, 25% glycerol, and 2.5% 1,4-Diazabicyclo(2.2.2)octane (all from Sigma-Aldrich) was added per well and covered with glass coverslips. Direct immunofluorescence of fixed cells was performed either with TRA-1-60-AF488 (1:10), or CD144-FITC (1:100) and CD34-APC (1:500), or CD43-FITC (1:100) and CD34-APC (1:500) diluted in 2% BSA, 0.2% Triton X-100 in DPBS. CD34-APC alone was also employed 1:500 in 0.1% BSA in DPBS, omitting permeabilization to prevent loss of mKO2 fluorescence. Pictures were acquired by sequential scan on a Leica TCS SP8X confocal microscope equipped with a 405 nm diode for DAPI and a white light laser (490 nm excitation for AF488 or FITC, 550 nm for mKO2 and 650 nm for APC) and an HC PL APO CS2 40x/1.10 water immersion objective. For live cell imaging, microphotographs were acquired at 35 °C. Antibodies are listed in Supplementary Table S2.

### 3.8. Flow Cytometry

For surface staining, live supernatant cells were incubated with an antibody cocktail (CD14-APC-Cy7, CD16-BV605, CD34-APC, and CD43-BV510) and analyzed on an LSRFortessa cytometer (Becton Dickinson; 405 nm excitation, 525 ± 50 nm and 605 ± 12 nm emission; 561 nm excitation and 581 ± 15 nm emission; 640 nm excitation, 670 ± 14 nm and 780 ± 60 nm emission).

For phosphoflow staining cells were starved for 4 h at 37 °C in RPMI-1640 medium without any supplements and fixed with 2% methanol-free formaldehyde for 15 min at RT. After centrifugation, cells were permeabilized with methanol for 30 min at −20 °C, incubated with pSTAT5 or isotype control antibody (1:50 in 0.1% BSA/DPBS), and fluorescence was measured for mKO2 (561 nm excitation and 581 ± 15 nm emission) and AlexaFluor647 (640 nm excitation and 670 ± 14 nm emission). In starved Ba/F3 cells, pSTAT5 was detected as described above, while V5-tagged RUNX1-JAK2 was detected separately with primary anti-V5 (1:1000) and secondary antimouse-IgG-AlexaFluor-488 antibodies (1:2000; 488 nm excitation and 530 ± 30 nm emission). Intact single cells were gated according to forward and sideward scatter and analyzed using FlowJo 10.5.2. Antibodies are listed in Supplementary Table S2.

## 4. Conclusions

We have established an in vitro model system, which allows interrogation of the impact of leukemia-associated fusions on hematopoietic differentiation and in a proof-of-principle study investigated RUNX1-JAK2. Following insertion of the fusion into one endogenous *RUNX1* allele of hiPSCs, we observed a decrease in hematopoietic progenitor output, which is most likely attributable to *RUNX1* haploinsufficiency. Expression of the RUNX1-JAK2 fusion protein led to constitutive STAT5 phosphorylation, but did not elicit significant effects on clonogenicity. However, RNA-seq analyses of RUNX1-JAK2-expressing hematopoietic cells revealed significant upregulation of genes related to the JAK-STAT and MYC pathways. In summary, the described combination of precise knock-in, hematopoietic differentiation of isogenic hiPSC lines, and targeted fusion protein degradation represents a versatile well-controlled approach to study oncogenic mechanisms in leukemia development.

## Data Availability

Gene expression profiling data have been deposited into the NCBI Gene Expression Omnibus database (accession number GSE159261).

## Supplementary Materials

The following Supplementary data are available online at https://www.mdpi.com/article/10.3390/ijms22147576/s1.
