# Supplementary material for: Expression of RUNX1-JAK2 in Human Induced Pluripotent Stem Cell-Derived Hematopoietic Cells Activates the JAK-STAT and MYC Pathways
Source: Int J Mol Sci. 2021 Jul 15;22(14):7576. doi: 10.3390/ijms22147576 (PMC8304339; doi:10.3390/ijms22147576)
Supplement: Supplementary file 1 [file ijms-22-07576-s001.zip › SupplementaryMaterial.pdf]

Supplementary Material

Expression of RUNX1-JAK2 in Human Induced Pluripotent Stem Cell-Derived Hematopoietic Cells Activates the JAK-STAT and MYC Pathways

Klaus Fortschegger, Anna-Maria Husa, Dagmar Schinnerl, Karin Nebral and Sabine Strehl

St. Anna Children's Cancer Research Institute, Vienna, Austria

International Journal of Molecular Sciences 2021

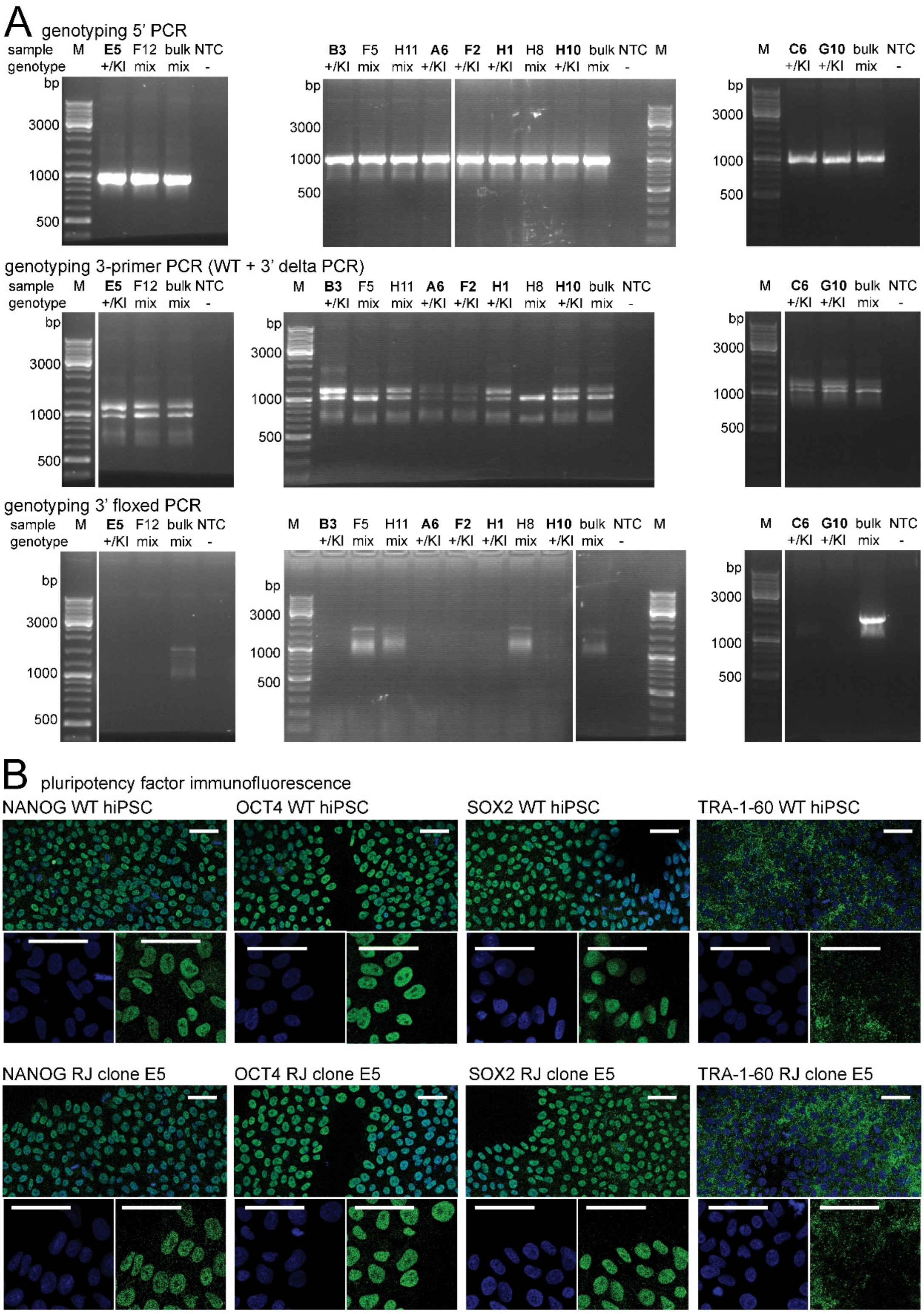

Supplementary Figure S1. Genotyping PCRs and pluripotency factor immunofluorescence (related to Figure 1D, E). (**A**) 5′ PCR, 3-primer PCR, and floxed 3′ PCR (upper, middle, and lower panels, respectively) of *RUNX1-JAK2* knock-in clones, cell bulk and no template controls (NTC) yield products of 965 bps for the 5′PCR and 1155 bps for the 3′ PCR of the recombined knock-in (KI) allele, 1010 bps for the WT PCR of the wild-type (+) allele, and 1535 bps for the floxed KI allele. Deduced genotypes are indicated (+, wild-type allele; KI, knock-in allele; mix, +/KI mixed with +/+ or +/floxed), pure heterozygous clones are marked in bold. (**B**) Immunofluorescence for pluripotency factors NANOG, OCT4, SOX2 and TRA-1-60 was performed with untreated WT hiPSCs and RJ clone E5 (upper and lower panels, respectively; indicated target in green; DAPI in blue; white bars = 50 µm). Non-overlaid close-ups (twofold magnification) are shown at the bottom.

5'HA (*RUNX1* intron 8) *JAK2* (exons 19 to 25) Linker-dTAG-HA IRES mKO2 loxP site 3'HA (*RUNX1* exon 9) SNP rs13051066

5′-ACCTCCTGGGACATGCAAAAATGCCAGATTTGGCTGGCAGGCTGCAGTTTGCCAACCCTAGGATTATATCAACATTTTAT
ATACGTTATACATATTATAGTATATACTATTGCACATTATATCTATACTATACATATTATGCTATACTACATCATTTCATATA
TCATGTCATATTACATTACATGATATTCTATATTACCGTATTATATTACTGTATATTATACCATCCTCCTGGGCAGTAGCAGC
CTCCTGATCCCCGTCCTCCTGGGCATAGCATCCTGGGTGACCTCATCTTTTTGGGTGGCAGATTCTGGGTAGAAACCCTCTCC
CTAGGCAGCAGCATCCTCAGTAGCCCCCATCCTCCCAGGTGGTAGCAATCCTGTGTGGTTCCAATCTTCCTGGGCGGTAAATT
CTGATAGAAACCCATCTCCCTGGACAGTAGCATCCTGGGTGTCCCCCGTCCTCCTAGGCGGTAGCATCCTGGGTGGTCTCCAT
CCTCCCAGGTGGTGTCATCCTGGGTGGTCTCCGTCCTCTCAGGAGGTGGCATCCTGAGTGGTCCCCGACCTCCTGGGCATAGC
ATCATGGGTAGTCCCCATCCTCTTGGGAGGTGACATGCTGGGTGATCCTCGTCATCTCAGGAGGTGGCATCCTGGGTGGTCCC
TGTCCCCCTGGGTATAGCATCCTGGGTAATCCTCGTCCTCTTGGGAGTAGCATCCCGGGTGGTCCCCGTCCTCCCCGGCAGTA
GCATCCTGGGTGGCTTCCCATCCTCCTAGGCGGTATCATCCTGGGTAGCCCCCTGGGGCAGAGGGAAGAGCTGTGGCCTCCGC
AACCTCCTACTCACTTCCGCTCCGTTCTCTTGCCCGCCCTGCAGATTATGAACTATTAACAGAAAATGACATGTTACCAAATA
TGAGGATAGGTGCCCTGGGGTTTTCTGGTGCCTTTGAAGACCGGGATCCTACACAGTTTGAAGAGAGACATTTGAAATTTCTA
CAGCAACTTGGCAAGGGTAATTTTGGGAGTGTGGAGATGTGCCGGTATGACCCTCTACAGGACAACACTGGGGAGGTGGTCGC
TGTAAAAAAGCTTCAGCATAGTACTGAAGAGCACCTAAGAGACTTTGAAAGGGAAATTGAAATCCTGAAATCCCTACAGCATG
ACAACATTGTAAAGTACAAGGGAGTGTGCTACAGTGCTGGTCGGCGTAATCTAAAATTAATTATGGAATATTTACCATATGGA
AGTTTACGAGACTATCTTCAAAAACATAAAGAACGGATAGATCACATAAAACTTCTGCAGTACACATCTCAGATATGCAAGGG
TATGGAGTATCTTGGTACAAAAAGGTATATCCACAGGGATCTGGCAACGAGAAATATATTGGTGGAGAACGAGAACAGAGTTA
AAATTGGAGATTTTGGGTTAACCAAAGTCTTGCCACAAGACAAAGAATACTATAAAGTAAAAGAACCTGGTGAAAGTCCCATA
TTCTGGTATGCTCCAGAATCACTGACAGAGAGCAAGTTTTCTGTGGCCTCAGATGTTTGGAGCTTTGGAGTGGTTCTGTATGA
ACTTTTCACATACATTGAGAAGAGTAAAAGTCCACCAGCGGAATTTATGCGTATGATTGGCAATGACAAACAAGGACAGATGA
TCGTGTTCCATTTGATAGAACTTTTGAAGAATAATGGAAGATTACCAAGACCAGATGGATGCCCAGATGAGATCTATATGATC
ATGACAGAATGCTGGAACAATAATGTAAATCAACGCCCCTCCTTTAGGGATCTAGCTCTTCGAGTGGATCAAATAAGGGATAA
CATGGCTGGAGTCGACGGTGGCGGTGGCTCGGGCGGTGGTGGGTCGGGTGGCGGCGGATCTGGAGTGCAGGTGGAAACCATCT
CCCCAGGAGACGGGCGCACCTTCCCCAAGCGCGGCCAGACCTGCGTGGTGCACTACACCGGGATGCTTGAAGATGGAAAGAAA
GTTGATTCCTCCCGGGACAGAAACAAGCCCTTTAAGTTTATGCTAGGCAAGCAGGAGGTGATCCGAGGCTGGGAAGAAGGGGT
TGCCCAGATGAGTGTGGGTCAGAGAGCCAAACTGACTATATCTCCAGATTATGCCTATGGTGCCACTGGGCACCCAGGCATCA
TCCCACCACATGCCACTCTCGTCTTCGATGTGGAGCTTCTAAAACTGGAAGGCTCTGGCTACCCCTACGACGTGCCCGACTAC
GCCGGCTATCCGTATGATGTCCCGGACTATGCAGGCTAGTCGACAAGCTTGCCCCTCTCCCTCCCCCCCCCCTAACGTTACTG
GCCGAAGCCGCTTGGAATAAGGCCGGTGTGCGTTTGTCTATATGTTATTTTCCACCATATTGCCGTCTTTTGGCAATGTGAGG
GCCCGGAAACCTGGCCCTGTCTTCTTGACGAGCATTCCTAGGGGTCTTTCCCCTCTCGCCAAAGGAATGCAAGGTCTGTTGAA
TGTCGTGAAGGAAGCAGTTCCTCTGGAAGTTTCTTGAAGACAAACAACGTCTGTAGCGACCCTTTGCAGGCAGCGGAACCCCC
CACCTGGCGACAGGTGCCTCTGCGGCCAAAAGCCACGTGTATAAGATACACCTGCAAAGGCGGCACAACCCCAGTGCCACGTT
GTGAGTTGGATAGTTGTGGAAAGAGTCAAATGGCTCTCCTCAAGCGTATTCAACAAGGGGCTGAAGGATGCCCAGAAGGTACC
CCATTGTATGGGATCTGATCTGGGGCCTCGGTGCACATGCTTTACATGTGTTTAGTCGAGGTTAAAAAAACGTCTAGGCCCCC
CGAACCACGGGGACGTGGTTTTCCTTTGAAAAACACGATGATAATATGGCCACAACCATGGTGAGTGTGATTAAACCAGAGAT
GAAGATGAGGTACTACATGGACGGCTCCGTCAATGGGCATGAGTTCACAATTGAAGGTGAAGGCACAGGCAGACCTTACGAGG
GACATCAAGAGATGACACTACGCGTCACAATGGCCGAGGGCGGGCCAATGCCTTTCGCGTTTGACTTAGTGTCACACGTGTTC
TGTTACGGCCACAGAGTATTTACTAAATATCCAGAAGAGATACCAGACTATTTCAAACAAGCATTTCCTGAAGGCCTGTCATG
GGAAAGGTCGTTGGAGTTCGAAGATGGTGGGTCCGCTTCAGTCAGTGCGCATATAAGCCTTAGAGGAAACACCTTCTACCACA
AATCCAAATTTACTGGGGTTAACTTTCCTGCCGATGGTCCTATCATGCAAAACCAAAGTGTTGATTGGGAGCCATCAACCGAG
AAAATTACTGCCAGCGACGGAGTTCTGAAGGGTGATGTTACGATGTACCTAAAACTTGAAGGAGGCGGCAATCACAAATGCCA
AATGAAGACTACTTACAAGGCGGCAAAAGAGATTCTTGAAATGCCAGGAGACCATTACATCGGCCATCGCCTCGTCAGGAAAA
CCGAAGGCAACATTACTGAGCAGGTAGAAGATGCAGTAGCTCATTCCTAATCTAGAAACGATCATATTCAATAACCCTTAATA
TAACTTCGTATAATGTATGCTATACGAAGTTATTAGGTCCCTCTCGAGGCACCCGACCTGACAGCGTTCAGCGACCCGCGCCA
GTTCCCCGCGCTGCCCTCCATCTCCGACCCCCGCATGCACTATCCAGGCGCCTTCACCTACTCCCCGACGCCGGTCACCTCGG
GCATCGGCATCGGCATGTCGGCCATGGGCTCGGCCACGCGCTACCACACCTACCTGCCGCCGCCCTACCCCGGCTCGTCGCAA
GCGCAGGGAGGCCCGTTCCAAGCCAGCTCGCCCTCCTACCACCTGTACTACGGCGCCTCGGCCGGCTCCTACCAGTTCTCCAT
GGTGGGCGGCGAGCGCTCGCCGCCGCGCATCCTGCCGCCCTGCACCAACGCCTCCACCGGCTCCGCGCTGCTCAACCCCAGCC
TCCCGAACCAGAGCGACGTGGTGGAGGCCGAGGGCAGCCACAGCAACTCCCCCACCAACATGGCGCCCTCCGCGCGCCTGGAG
GAGGCCGTGTGGAGGCCCTACTGAGGCGCCAGGCCTGGCCCGGCTGGGCCMCGCGGGCCGCCGCCTTCGCCTCCGGGCGCGCG
GGCCTCCTGTTCGCGACAAGCCCGCCGGGATCCCGGGCCCTGGGCCCGGCCACCGTCCTGGGGCCGAGGGCGCCCGACGGCCA
GGATCTCGCTGTAGGTCAGGCCCGCGCAGCCTCCTGCGCCCAGAAGCCCACGCCGCCGCCGTCTGCTGGCGCCCCGGCCCTCG
CGGAGGTGTCCGAGGCGACGCACCTCGAGGGTGTCCGCCGGCCCCAGCACCCAGGGGACGCGCTGGAAAGCAAACAGGAAGAT
TCCCGGAGGGAAACTGTGAATGCTTCTGATTTAGCAATGCTGTGA-3′

Supplementary Figure S2. *RUNX1-JAK2* knock-in allele sequence. The relevant sequenced region of the final *RUNX1-JAK2* knock-in allele is shown with colored annotated regions.

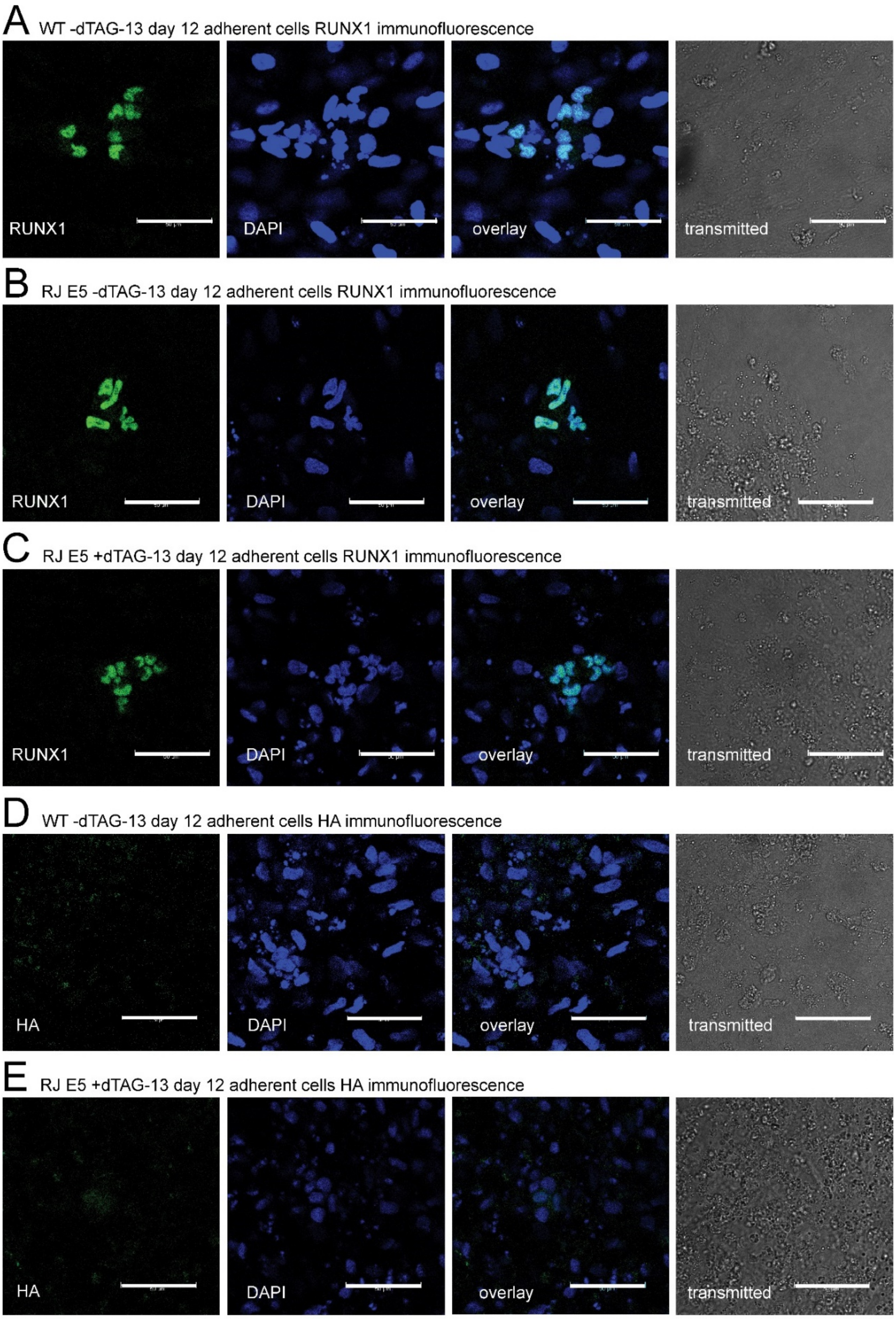

Supplementary Figure S3. RUNX1 and HA immunofluorescence. Indirect immunofluorescence control stainings (related to Figure 2D, E) were performed with unstarved adherent cells fixed after 12 days of differentiation. RUNX1 protein expression and localization (green) was determined in untreated wild-type (WT; **A**), and *RUNX1-JAK2* knock-in cells (RJ) untreated (**B**) or treated with 100 nM dTAG-13 (**C**); DAPI counterstain (blue), overlay, and transmitted light (grey) pictures are also shown. Untreated WT (**D**) and dTAG-13-treated RJ (**E**) cells were stained with an antibody raised against HA-tag (green) as negative controls. White bars correspond to 50 μm.

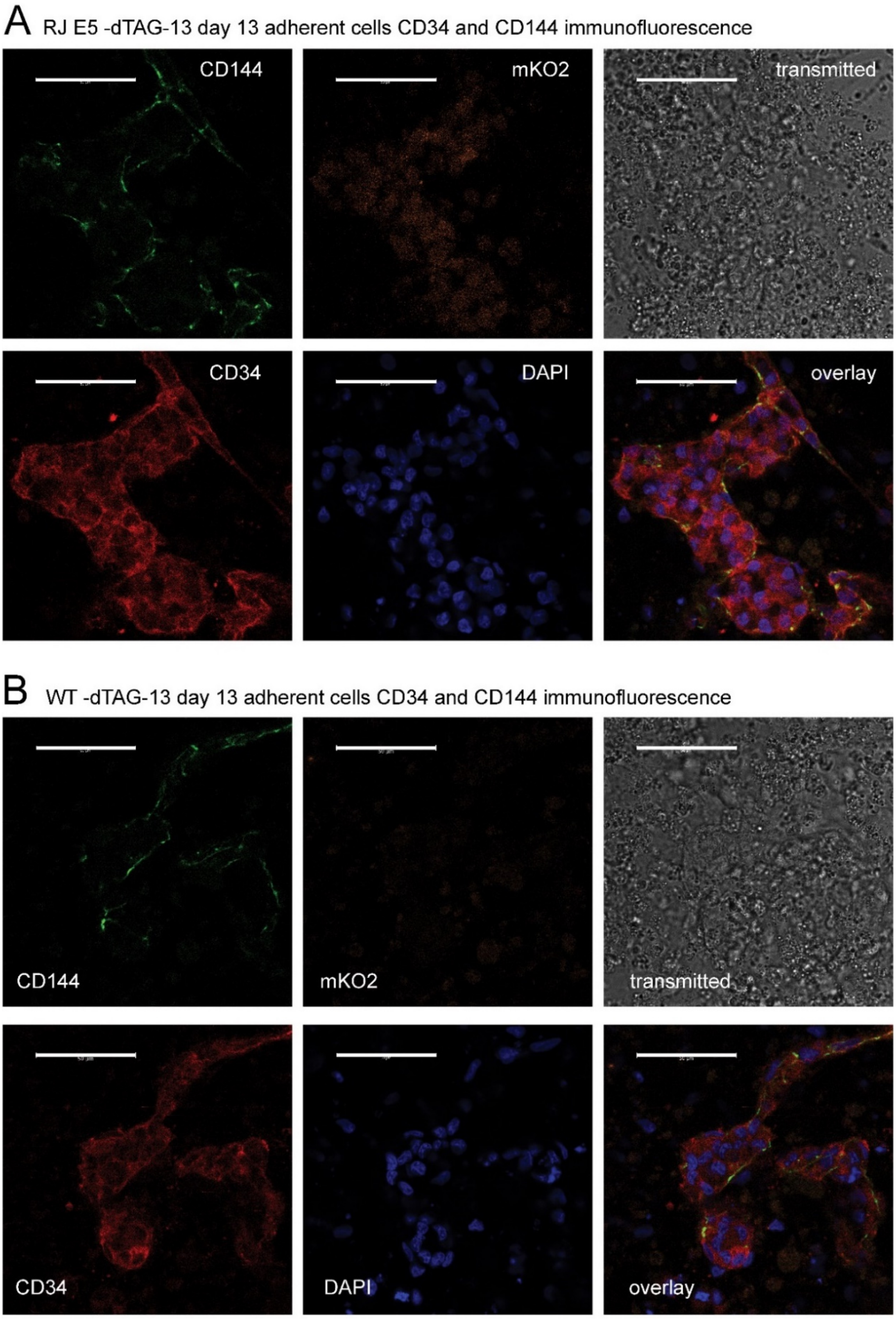

Supplementary Figure S4. CD34 and CD144 immunofluorescence (related to Figure 2F). Direct immunofluorescence co-stainings were performed with unstarved adherent cells fixed after 13 days of differentiation. Untreated *RUNX1-JAK2* knock-in (RJ; **A**) and wild-type (WT; **B**) cells were incubated with antibodies raised against vascular endothelial cadherin (CD144-FITC, green) and CD34 (APC, red); mKO2 fluorescence (orange), DAPI counterstain (blue), overlay, and transmitted light (grey) pictures are also shown. Triton X-100 detergent in the incubation buffer caused a significant decrease of mKO2 fluorescence. White bars correspond to 50 μm.

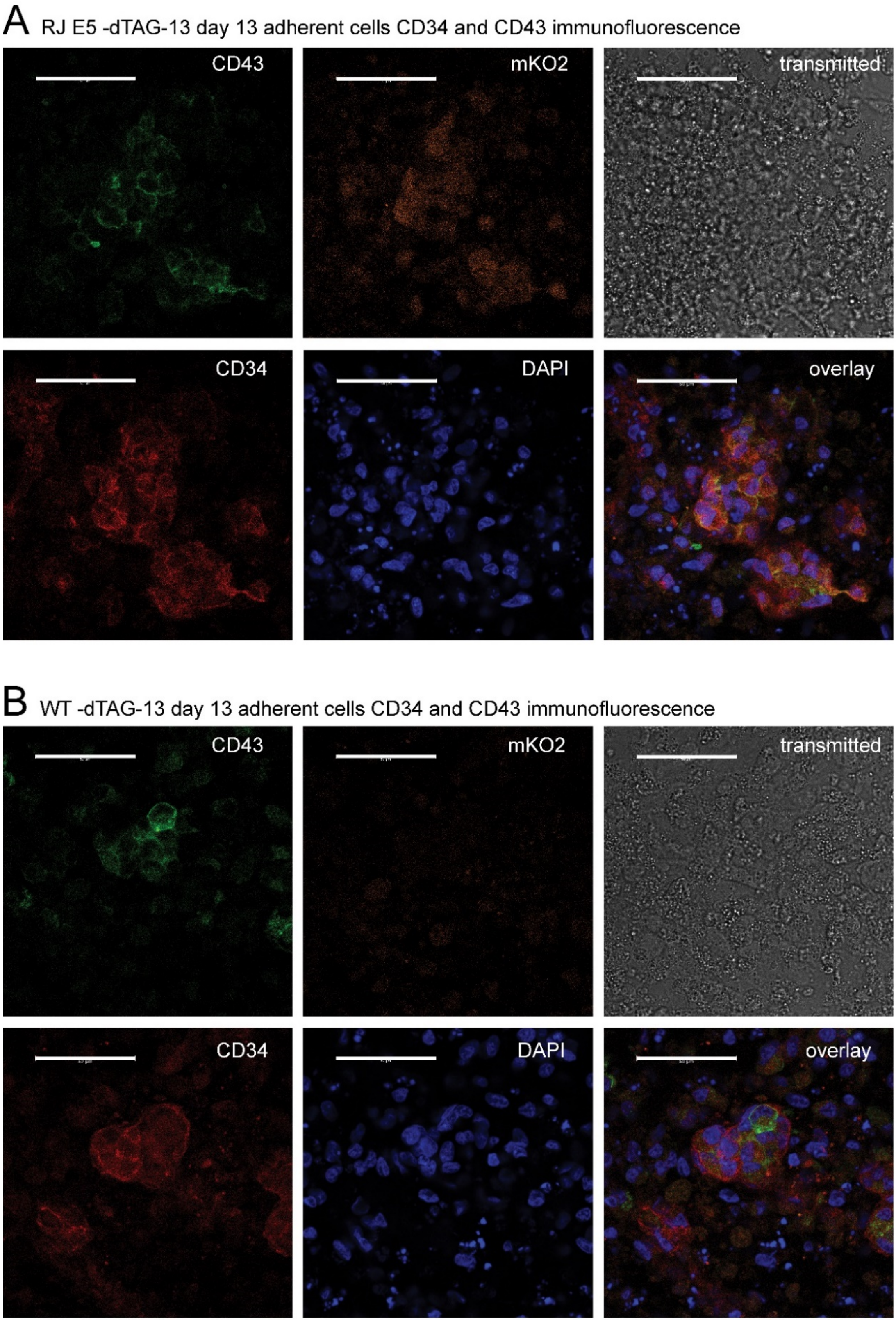

Supplementary Figure S5. CD34 and CD43 immunofluorescence (related to Figure 2F). Direct immunofluorescence co-stainings were performed with unstarved adherent cells fixed after 13 days of differentiation. Untreated *RUNX1-JAK2* knock-in (RJ; **A**) and wild-type (WT; **B**) cells were incubated with antibodies raised against leukosialin (CD43-FITC, green) and CD34 (APC, red); mKO2 fluorescence (orange), DAPI counterstain (blue), overlay, and transmitted light (grey) pictures are also shown. Triton X-100 detergent in the incubation buffer caused a significant decrease of mKO2 fluorescence. White bars correspond to 50 μm.

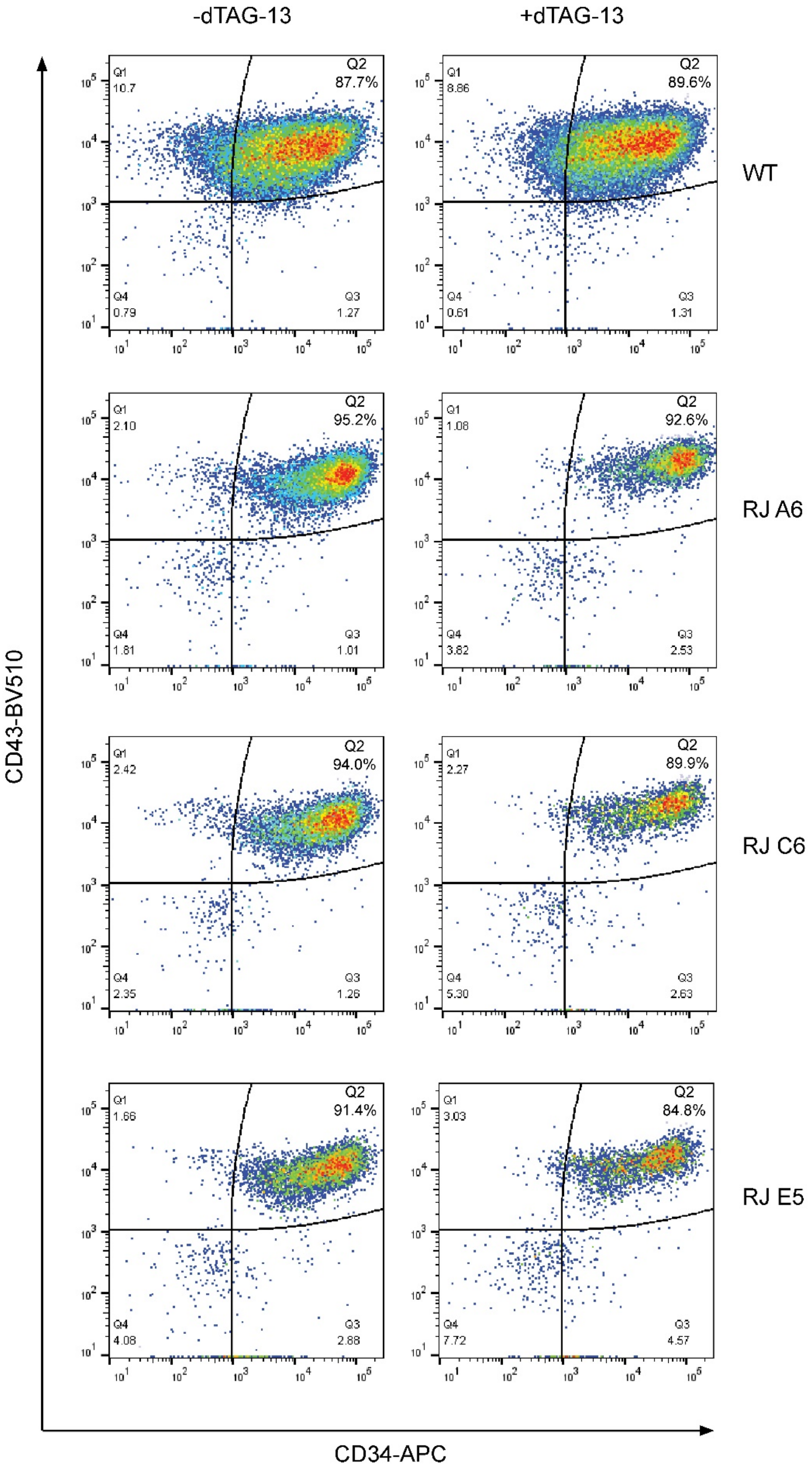

Supplementary Figure S6. CD34 and CD43 flow cytometry (related to Figure 3A). Supernatants of wild-type (WT) and RUNX1-JAK2 knock-in (RJ) clone cultures differentiated for 12 days with (right) or without (left) dTAG-13 were harvested, live cells were isolated, stained with antibody cocktail, and analyzed by flow cytometry. Intact single cells were gated according to forward and sideward scatter. CD34+ CD43+ (quadrant Q2) hematopoietic progenitor percentages are summarized in Figure 3A.

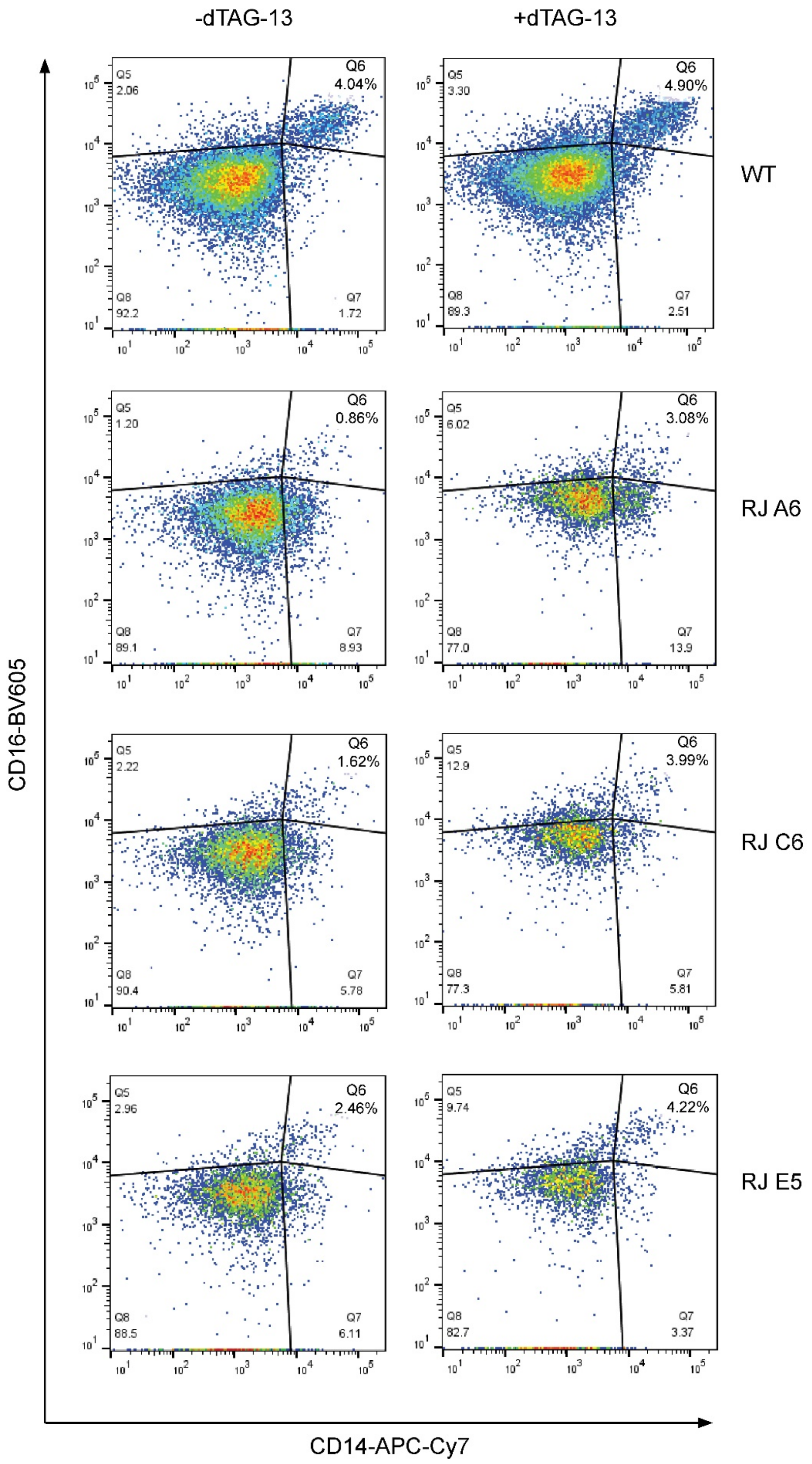

Supplementary Figure S7. CD14 and CD16 flow cytometry (related to Figure 3A). Supernatants of wild-type (WT) and RUNX1-JAK2 knock-in (RJ) clone cultures differentiated for 12 days with (right) or without (left) dTAG-13 were harvested, live cells were isolated, stained with antibody cocktail and analyzed by flow cytometry. Intact single cells were gated according to forward and sideward scatter. CD14+ CD16+ (quadrant Q6) monocyte percentages are summarized in Figure 3A.

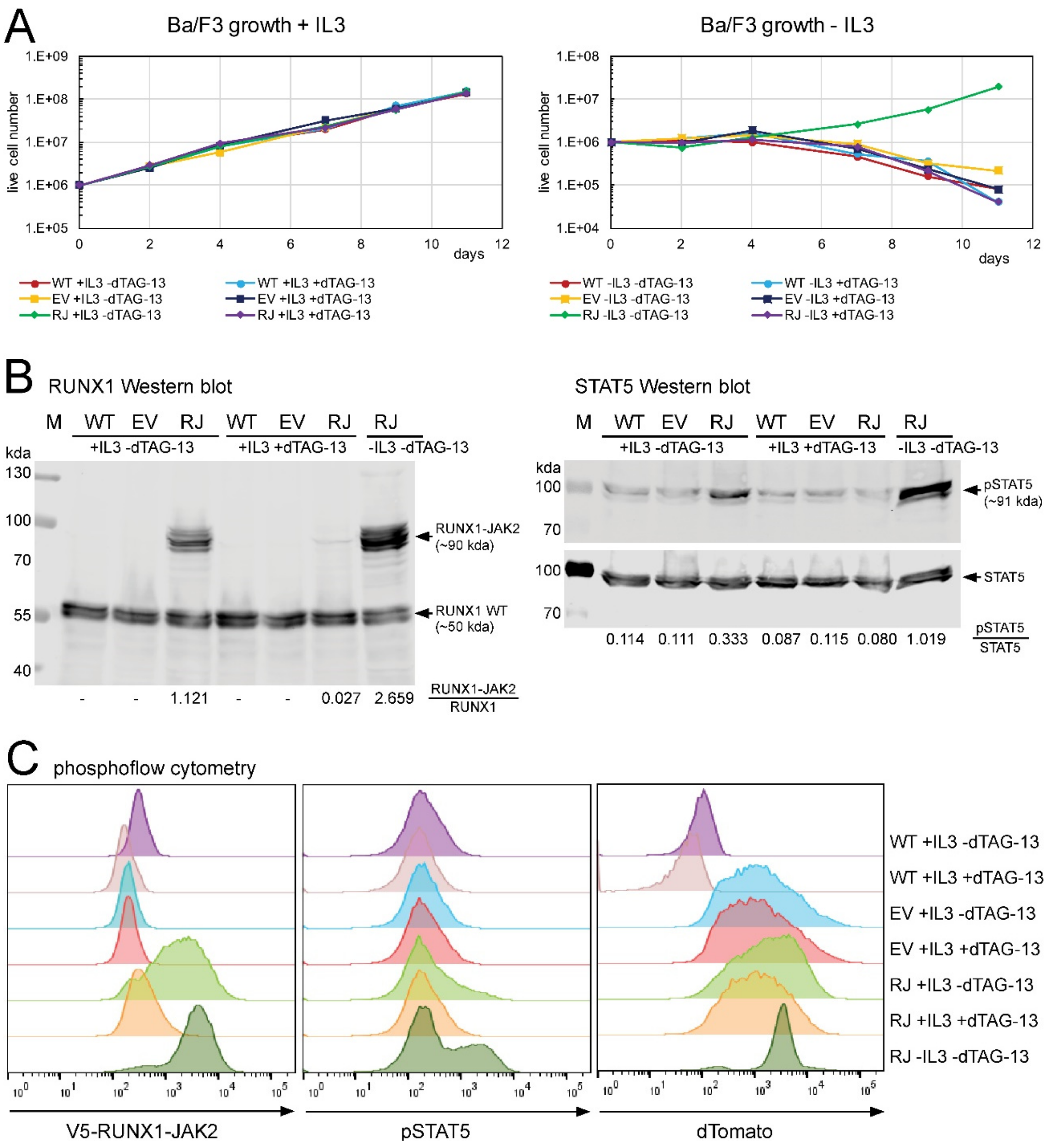

Supplementary Figure S8. RUNX1-JAK2 renders murine lymphoid Ba/F3 cells IL3-independent and induces STAT phosphorylation (related to Figure 3). Ba/F3 (kindly provided by Meinrad Busslinger, Institute for Molecular Pathology, Vienna, Austria) were grown in RPMI 1640 medium containing 10% fetal bovine serum (FBS), 100 U/ml penicillin, 100 µg/ml streptomycin (all from Gibco), with or without 10% IL3-medium conditioned by Wehi-3B cells (kindly provided by Herbert Strobl, Medical University of Graz, Austria), and with 100 nM dTAG-13 or DMSO vehicle only. Two million Ba/F3 cells were transfected in 100 µl solution V using Nucleofector 2b (Lonza) and program X-001, 4.5 µg transposon (pITR-ETP empty vector or V5-RUNX1-JAK2-dTAG-2xHA-containing) and 0.5 µg Sleeping Beauty 100xCo pcGlobin2 (vectors kindly provided by Eric Kowarz and Rolf Marschalek, Goethe-University, Frankfurt, Germany). Cell bulks with stably integrated transposons were selected with 1 µg/ml puromycin and exhibited >90% positivity for dTomato reporter. (**A**) One million cells (wild-type Ba/F3, WT; empty vector, EV; RUNX1-JAK2-containing, RJ) were seeded into media +/- IL3 and +/- dTAG-13 and growth monitored for 11 days. (**B**) Proliferation-competent cell bulks were starved for 3 h in RPMI 1640 and RUNX1-JAK2 to RUNX1 (left) as well as pSTAT5 to STAT5 protein ratios (right) were determined by Western blot with RUNX1 and STAT5 antibodies, respectively. (**C**) In addition, starved single cell bulks were subjected to flow cytometry to analyze the distribution of V5-tagged RUNX1-JAK2, pSTAT5 and dTomato protein expression. The results suggest that cells expressing high levels of RUNX1-JAK2 were selected by IL3 deprivation because in this case constitutively active JAK-STAT signaling allows for survival and proliferation.

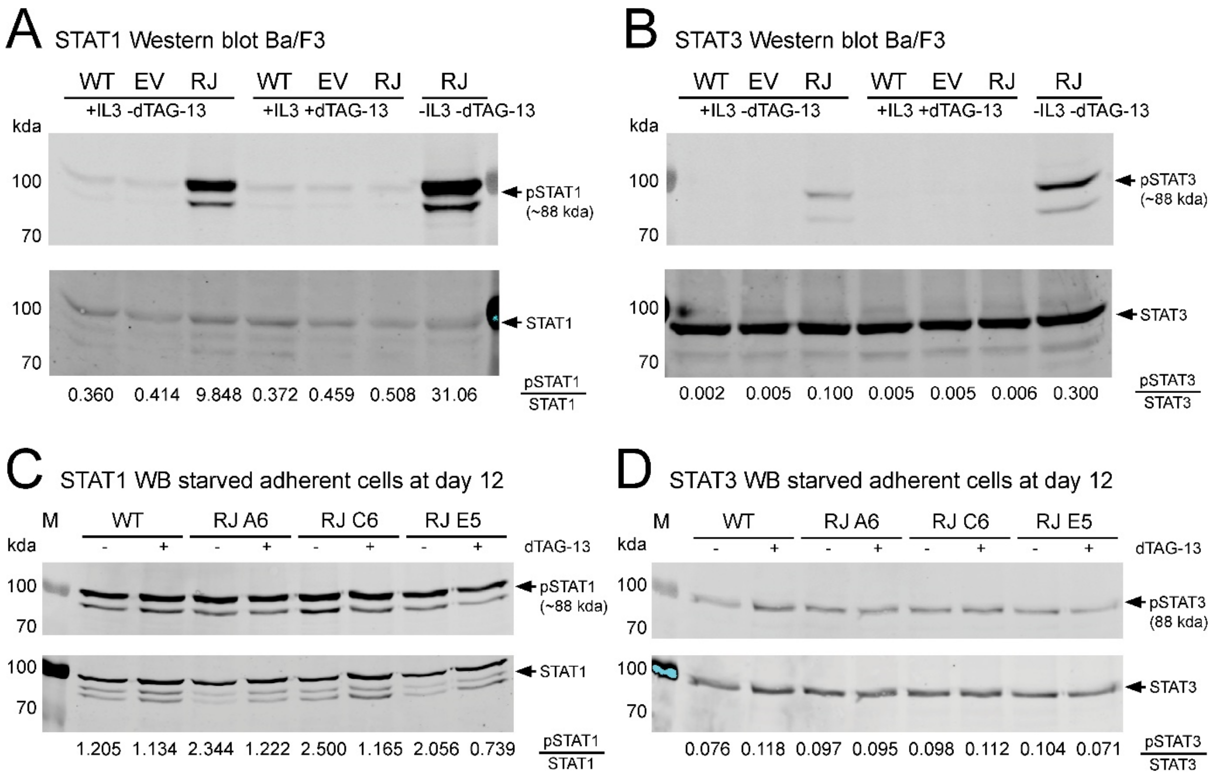

Supplementary Figure S9. Western blots for pSTAT1 (**A**) and pSTAT3 (**B**) were performed with starved Ba/F3 cells as previously described. Western blots (WB) for pSTAT1 (**C**) and pSTAT3 (**D**) were also performed with starved adherent wild-type (WT) and RUNX1-JAK2 (RJ) knock-in hiPSCs differentiated for 12 days.

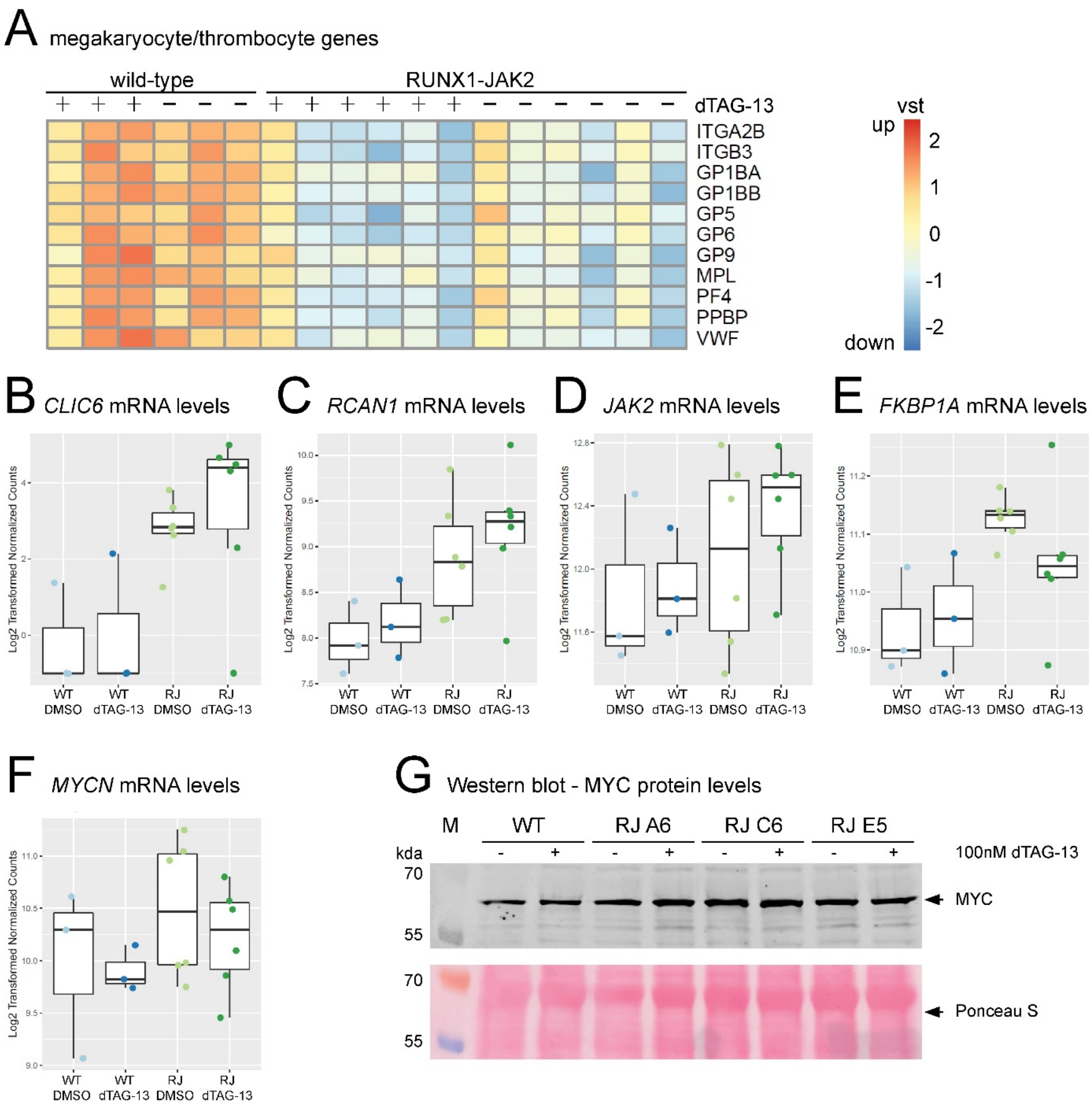

Supplementary Figure S10. Additional gene expression data (related to Figure 4). RNA-seq was conducted with wild-type (WT, n=3) and RUNX1-JAK2 (RJ, n=6) cells differentiated for 12 days either with (+, dTAG-13) or without (-, DMSO) degrader treatment. (**A**) Heatmap showing batch-corrected and variance-stabilization-transformed (vst) log_2-fold changes from low (blue) to high expression (orange) levels of selected significantly regulated megakaryocyte genes. Boxplots showing normalized log_2-transformed mRNA expression levels for *CLIC6* (**B**), *RCAN1* (**C**), *JAK2* (**D**), *FKBP1A* (**E**) and *MYCN* (**F**). (**G**) Western blot of dTAG-13-treated or untreated WT and three clonal RJ lines (unstarved adherent cell lysates at day 12) was performed for MYC protein. Ponceau S staining is shown below as loading control.

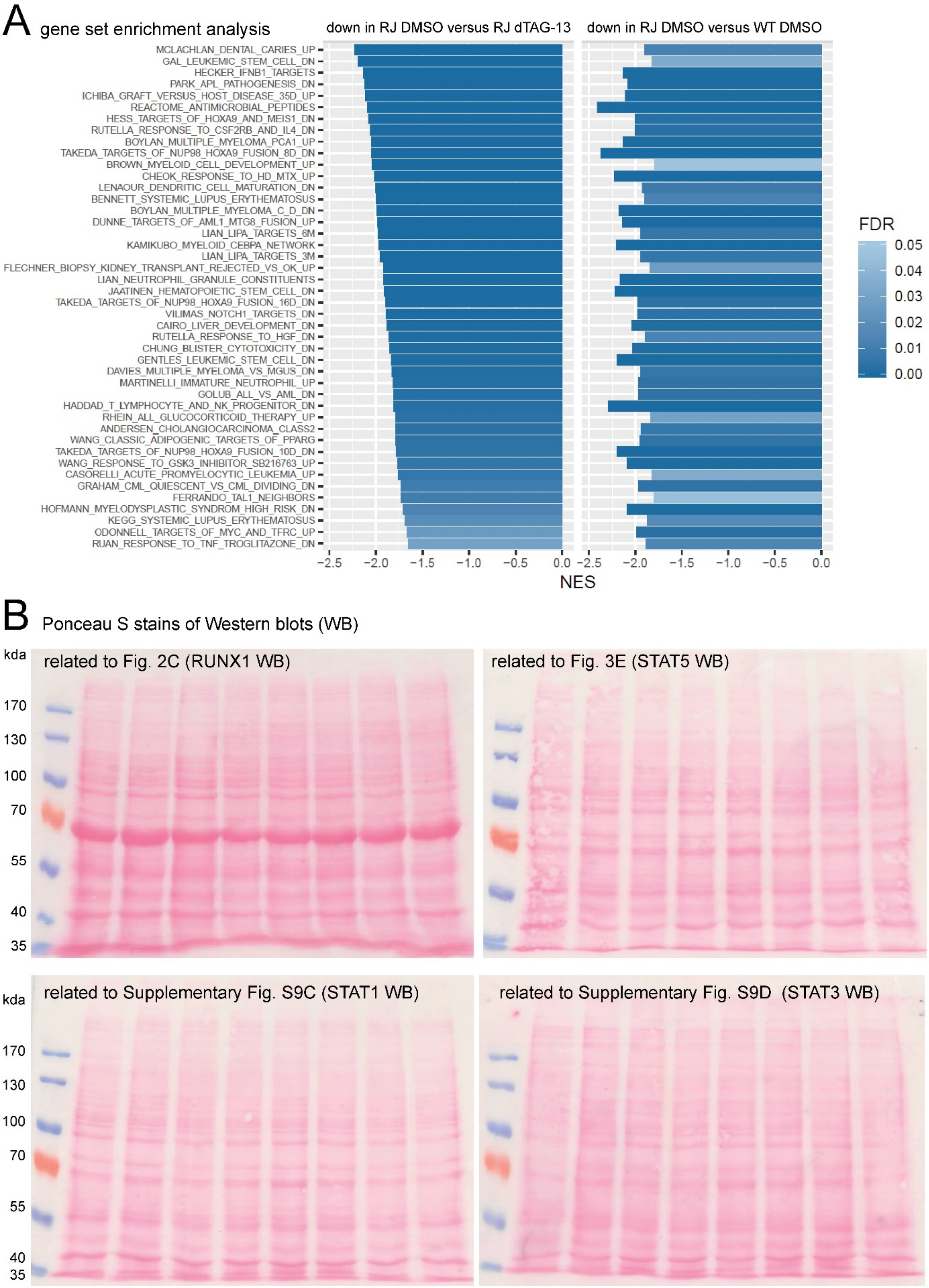

Supplementary Figure S11. Gene set enrichment analysis results (related to Figure 4) and Western blot loading controls. (**A**) Gene sets significantly downregulated (FDR q-values ≤ 0.05) with both comparisons, untreated RJ versus dTAG-13-treated RJ as well as untreated RJ versus untreated WT [the x-axis represents normalized enrichment scores (NES), the brightness of the bar q-values (FDR), respectively]. (**B**) Loading controls of Western blots with differentiated hiPSCs (related to Figures 2, 3, and Supplementary Figure S9).

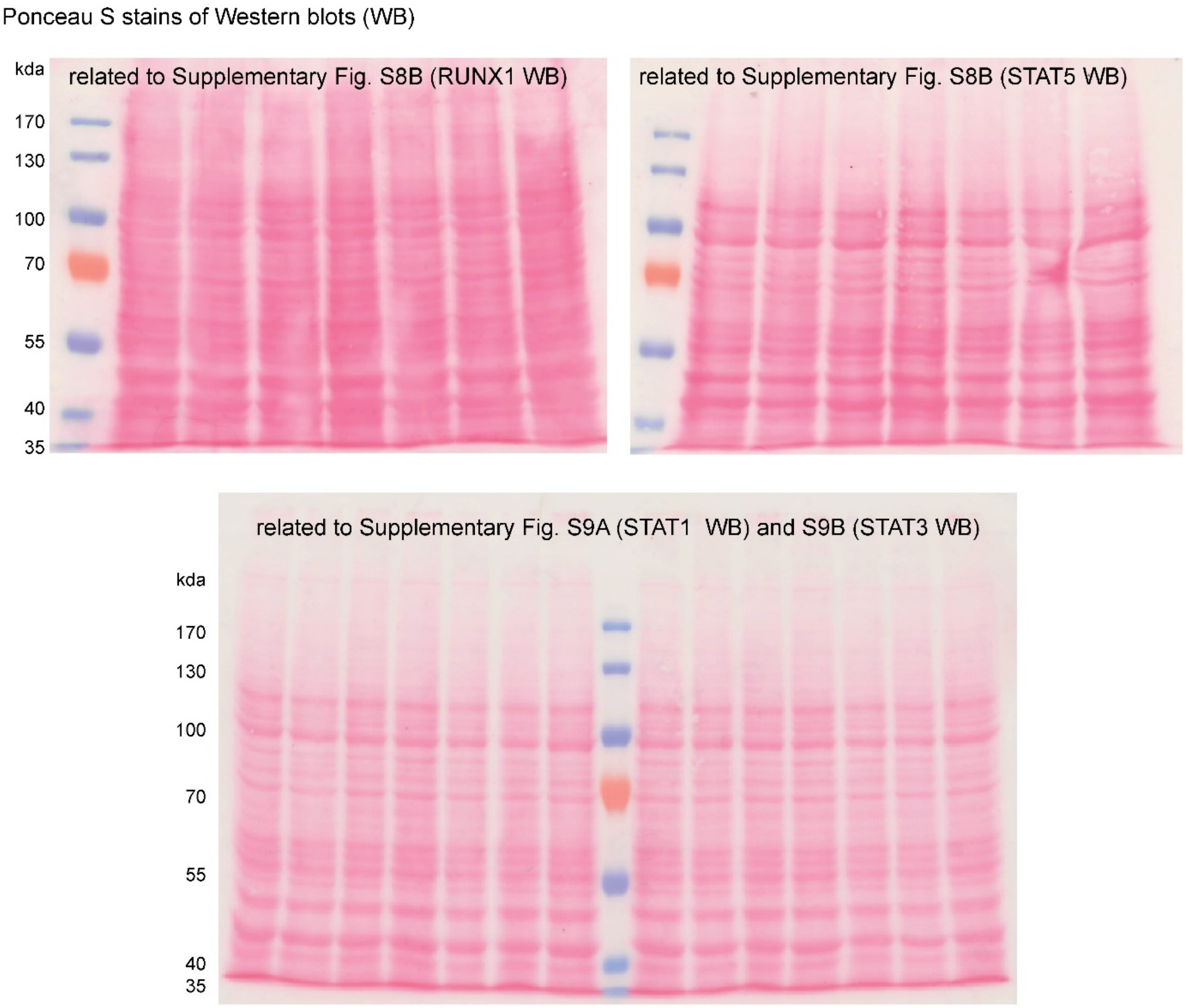

Supplementary Figure S12. Ba/F3 Western blot loading controls (related to Supplementary Figure S8, S9).

Supplementary Table S1. Detailed RNA-seq results (separate Excel file)

RNA-seq differential expression of 15615 transcripts with base mean expression > 6.078. **Panel 1**: columns A-C: gene identifiers; column D: base mean normalized expression; columns E-H: shrunken log_2-transformed fold changes of indicated comparisons (fold changes ≥ 2 highlighted by colored background); columns I-L: adjusted p-values of indicated shrunken fold changes (p-values ≤ 0.01 highlighted by colored background); columns M-AD: DESeq2-normalized expression values for each sample. **Panels 2-7**: GSEA results: up- and downregulated gene sets for given comparisons. **Panel 8**: RNA-seq stats.

Supplementary Table S2. Material lists (separate Excel file)

Panel 1: antibody list. **Panel 2:** oligonucleotide list.
